# Depolymerization-Induced Morphological Transformation

**DOI:** 10.1021/jacs.5c18937

**Published:** 2026-01-26

**Authors:** Nethmi De Alwis Watuthanthrige, Victoria Lohmann, Viviane Lutz-Bueno, Nghia P. Truong, Steven P. Armes, Athina Anastasaki

**Affiliations:** † Laboratory for Sustainable Polymers, Department of Materials, 27219 ETH Zurich, Vladimir-Prelog-Weg 5, 8093 Zürich, Switzerland; ‡ PSI Center for Neutron and Muon Sciences, 5232 Villigen PSI, Switzerland; § School of Mathematical and Physical Sciences, Dainton Building,University of Sheffield, Sheffield, South Yorkshire S3 7HF, U.K.

## Abstract

Depolymerization offers a powerful route for the chemical recycling of vinyl polymers. However, current strategies focus almost exclusively on monomer recovery, which overlooks broader applications and opportunities. Herein, depolymerization-induced morphological transformation (DIMT) is introduced as a modular methodology to control the shape of sterically stabilized diblock copolymer nanoparticles and gain mechanistic insight into morphological transformations that occur during selective degradation of the methacrylic core-forming block. Notably, DIMT results in a sequential evolution in copolymer morphology from vesicles to worms to spheres. Transmission electron microscopy (TEM) and small-angle X-ray scattering (SAXS) studies enabled the construction of a predictive (pseudo)­phase diagram. Furthermore, this new approach was also applied to the irreversible degelation of diblock copolymer worm gels, highlighting new opportunities to regulate material properties through depolymerization.

## Graphical abstract

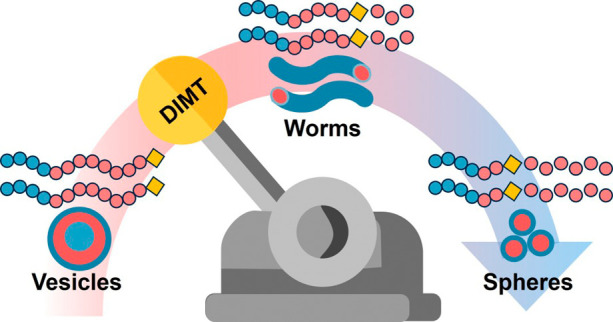

## Introduction

Chemical recycling to monomer not only enables an ideal circular polymer economy but also bypasses product deterioration often caused by alternative methodologies, such as mechanical recycling.^1^ However, the feasibility of efficient depolymerization is highly dependent on the polymer structure: materials containing cleavable heteroatoms in the backbone are more prone to bond cleavage and degradation, while vinyl polymers containing highly stable carbon–carbon backbones present additional challenges.^2^ Pyrolysis represents the most traditional industrial approach to depolymerizing vinyl polymers. However, it requires high temperatures (typically above 400 °C) and often results in a complex mixture of byproducts.^3^ To overcome these challenges, milder depolymerization strategies have recently been explored, exploiting the high end-group fidelity introduced by controlled radical polymerization (CRP) techniques.^4^ In particular, the incorporation of labile chain-ends such as thiocarbonylthio groups in reversible addition–fragmentation chain transfer (RAFT) polymerization and halogens in atom transfer radical polymerization (ATRP) has enabled efficient monomer recovery at temperatures ranging from 90 to 170 °C.^5^ Through either thermal or photothermal depolymerization methodologies,^4,6,7^ the groups of Gramlich,^8^ Sumerlin,^9–14^ Ouchi,^15^ Matyjaszewski,^16–20^ our group,^21–36^ and others^37–41^ have pioneered the chemical recycling of polymers containing both bulky and nonbulky side chains resulting in high depolymerization yields, often exceeding 80–90%. The vast majority of these strategies rely on the presence of functional chain-ends to recover monomer at lower temperatures, limiting further applications and scope.

However, the well-defined end-groups that are characteristic of CRP also enable precise control over the mean degree of polymerization (DP) of each block (an essential parameter in self-assembly), thereby creating an opportunity to direct the formation and shape of block copolymer nanoparticles.^42,43^ For example, polymerization-induced self-assembly (PISA) is a robust and highly versatile technique that enables the synthesis of diblock copolymer nanoparticles typically consisting of three main morphologies: spheres, worms, and vesicles.^44–46^ These morphologies have been evaluated for a wide range of applications, including catalysis, sensing, imaging, energy storage, tissue engineering, and targeted drug delivery.^47–50^ The transition between the different morphologies is governed by multiple parameters including molecular weight, solids content, and polymer end-groups.^51,52^ The relative volume fraction, which is directly related to the molecular weight of each block, influences both the size and shape of the resulting nanoparticles, as predicted by the geometric packing parameter theory.^53^ A gradual increase in molecular weight of the insoluble structure-directing block typically drives morphological transitions from spheres to worms to vesicles.^54^ However, several reports have also demonstrated the effect of copolymer concentration on nanoparticle morphology, so a comprehensive mechanistic understanding of the morphological transformation during PISA has not been fully elucidated.^54–56^ Furthermore, in current PISA formulations of vinyl polymers, morphological transitions cannot be achieved by simply reducing the DP of the insoluble block; instead, such transitions typically require chemical modification of the polymer structure to change its amphiphilicity or block incompatibility.^57–59^ A modular depolymerization strategy could not only enable morphological control purely through molecular weight reduction but also offer useful mechanistic insights into morphological transitions. In this context, an inspiring study by Seo and co-workers recently explored entropy-driven depression of the ceiling temperature (*T*_*c*_) to regulate the polymerization–depolymerization equilibrium in situ during the ring-opening polymerization (ROP) of δ-valerolactone.^60^ This approach enabled polymeric materials containing heteroatoms to undergo rod-to-sphere or fiber-to-rod transitions. However, such morphological changes were restricted to specific compositions of poly­(ethylene oxide)*-b-*poly­(δ-valerolactone) (PEO*-b-*PVL), with no significant transformation observed for copolymers comprising shorter PEO blocks. Importantly, this pioneering study raises the question of whether morphological transitions between spheres, worms, and vesicles might be induced via selective depolymerization of the core-forming block.

Herein, we introduce the first example of depolymerization-induced morphological transformation (DIMT) of vinyl polymers, which results in the sequential evolution of vesicles to worms to spheres, as shown in Scheme 1. Notably, each of these three morphologies can be obtained in high purity from a single starting material simply by regulating the DP of the insoluble structure-directing block.

**Scheme 1:**
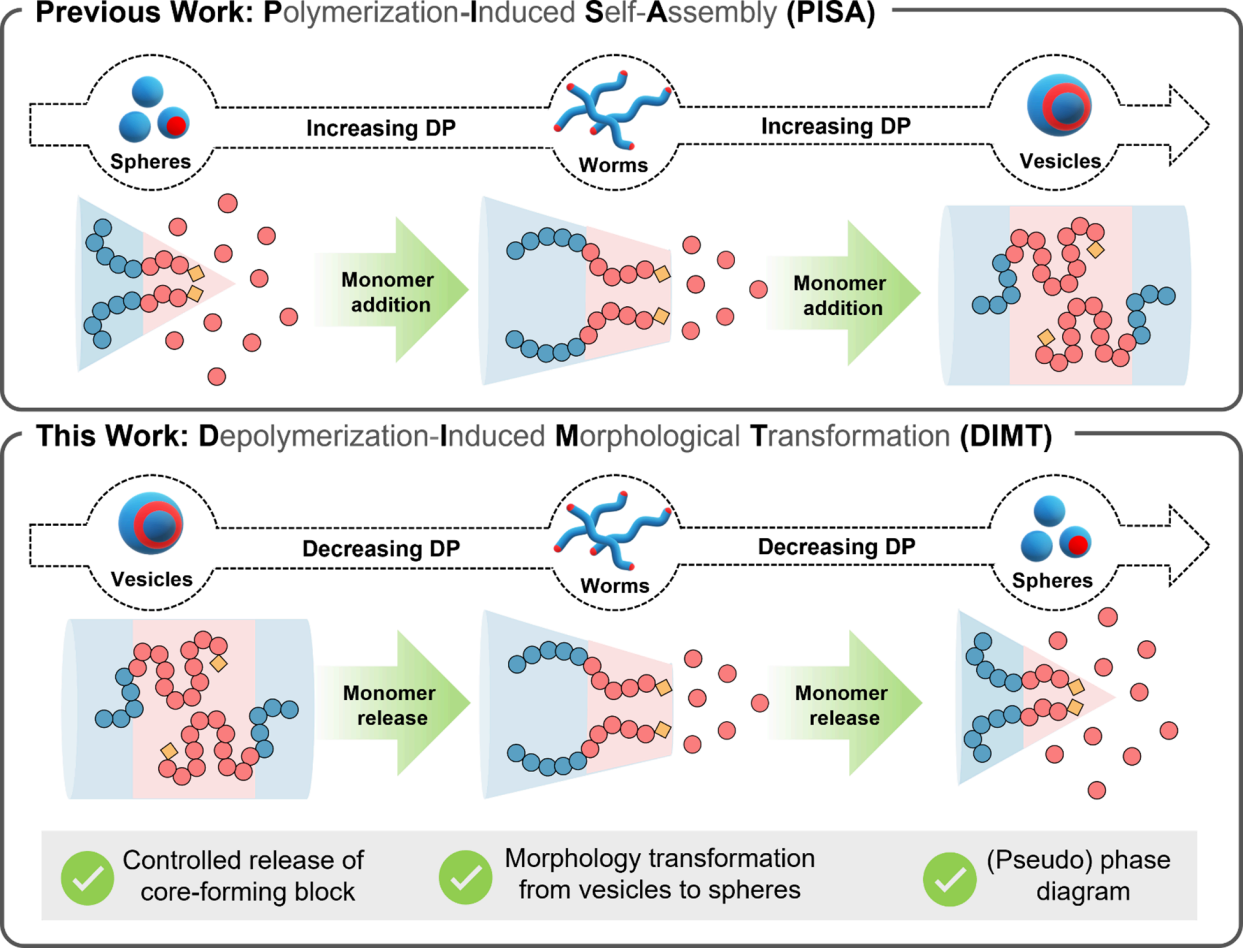
Conceptual Scheme Showing the Principles of PISA and DIMT

## Results and Discussion

In principle, an ideal DIMT process should provide access to each of the three main copolymer morphologies reported for PISA syntheses. To meet these criteria, poly­(lauryl methacrylate)*-b-*poly­(benzyl methacrylate) (PLMA*-b-*PBzMA) was selected as a suitable diblock copolymer system (Figure 1a).^61^ The PLMA precursor was synthesized using 2-cyano-2-propyl benzodithioate as the chain transfer agent (CTA) for the RAFT homopolymerization of LMA in toluene at 80 °C, initially targeting a DP of 16 (Figures S1 and S2, and Table S1). Subsequent chain extension with BzMA (targeting 20 wt % solids in n-dodecane) led to the formation of sterically stabilized PLMA*-b-*PBzMA nanoparticles, and the reaction was allowed to proceed until the BzMA conversion exceeded 95%. ^1^H NMR spectroscopy studies indicated the synthesis of PLMA_16_ *-b-*PBzMA_72_ nanoparticles, while TEM analysis confirmed the formation of vesicles (Figure S3). To trigger an efficient yet selective depolymerization suitable for demonstrating the predicted sequential morphology transformations, the solution was diluted to a repeat unit concentration of 25 mM (which corresponds to 0.7 wt % solids in n-dodecane), and the reaction temperature was raised to 100 °C, which corresponds to relatively mild conditions compared to previous reports.^4^ However, this only resulted in the depolymerization of 15% of the BzMA repeat units (Figure S4). Thus, a suitable high temperature radical initiator, 1,1′-azobis­(cyclohexanecarbonitrile) (ABCN, 10-h half-life at 88 °C,^62^ 0.6 equiv with respect to the polymer chain-end) was added to achieve a higher monomer yield with good selectivity, while maintaining a sufficiently slow rate of depolymerization to enable the in situ evolution in copolymer morphology to be monitored. The initiator loading proved to be critical; reducing ABCN below 0.6 equiv significantly reduced the extent of BzMA regeneration (Figure S4), which is likely due to insufficient initiator incorporation within the nanoparticle cores for the effective activation of chain-ends.

**Figure 1:**
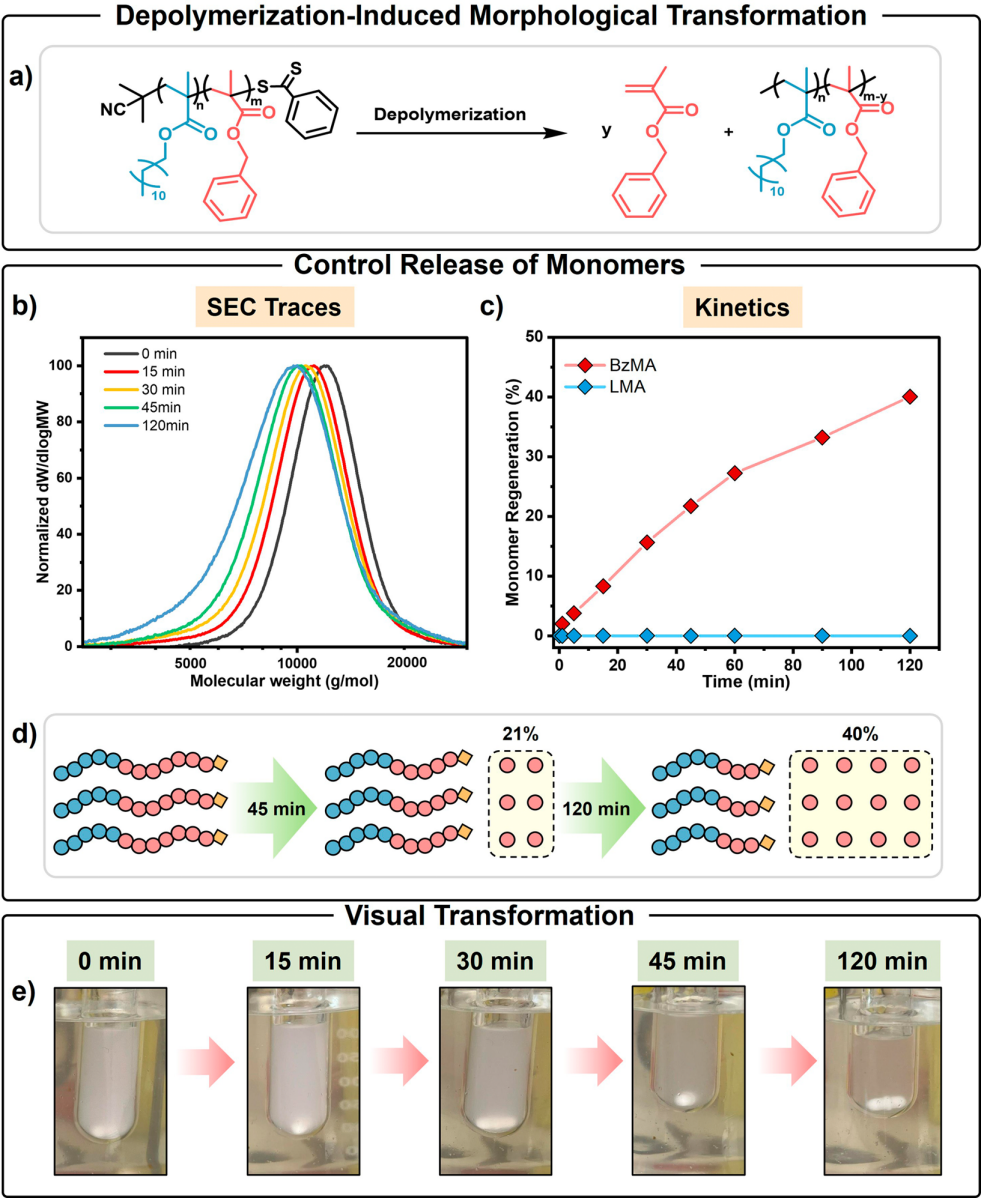
DIMT: (a) reaction scheme of the controlled generation of BzMA monomer from a PLMA*-b-*PBzMA diblock polymer, (b) corresponding SEC traces for this depolymerizing formulation, (c) selective regeneration of BzMA monomer over time (analyzed using ^1^H NMR), (d) schematic representation of the controlled generation of BzMA monomer, and (e) visual transformation of the reaction mixture at various time points during depolymerization of the PBzMA block.

Under these optimized conditions, 8% of BzMA monomer was generated within 15 min, as determined by ^1^H NMR analysis. Accordingly, the resulting diblock composition was calculated to be PLMA_16_ *-b-*PBzMA_66_ based on NMR analysis (Figure S5, Table S2), which was supported by the concomitant reduction in copolymer molecular weight indicated by SEC analysis (Figure 1b, Table S3). Notably, no release of LMA was detected at this early stage of depolymerization (Figure 1c), indicating highly selective and controlled generation of monomer from the core-forming block alone (Figure 1d). This partial depolymerization was accompanied by a transition from pure vesicles to a binary mixture of vesicles and worms (Figure S6a). As the BzMA depolymerization continued, analysis of aliquots extracted after 30 or 45 min revealed that the mixed phase of vesicles and worms persisted after generating 15% and 21% BzMA respectively, which corresponded to a further reduction in the molecular weight of the core-forming block to yield PLMA_16_-*b*-PBzMA_61_ and PLMA_16_-*b*-PBzMA_57_. The BzMA recovery increased to 40% within 2 h, which produced a morphological transition to afford PLMA_16_ *-b-*PBzMA_43_ worms. This vesicle-to-worm transition was verified by both visual inspection and subsequent characterization. First, the copolymer dispersion gradually changed from opaque (vesicles) to relatively transparent (worms, Figure 1e). Additionally, dynamic light scattering (DLS) measurements indicated a significant reduction in nanoparticle diameter during depolymerization (Figure S6b). The kinetics of depolymerization were monitored by ^1^H NMR spectroscopy, which revealed the controlled generation of solely BzMA monomers, suggesting that the observed molecular weight reduction exclusively involved the core-forming PBzMA block (Figure S6c). Crucially, a control experiment (thermal annealing in air in the absence of any additional radical initiator) yielded no discernible change in the copolymer morphology, confirming that the observed transitions are driven exclusively by depolymerization of the core-forming block (Figure S7) rather than the elevated temperature alone. This represents a significant improvement in selectivity for the second block over previous reports of controlled depolymerization, whereby vinyl proton signals corresponding to both monomers were observed within 15 min at 120 °C, despite the use of excess CTA (20 times) or high polymer concentrations (100 mM).^31^

Collectively, these observations indicate that the morphological transition is primarily driven by the reduction in the mean DP of the PBzMA block, which lowers the critical packing parameter and favors the formation of the lower-order morphology (i.e., worms). Moreover, the generated BzMA monomer is expected to plasticize the remaining PBzMA chains, thus increasing their mobility and aiding an evolution in copolymer morphology. Third, SEC analysis revealed a continuous reduction in molecular weight, with closely matching theoretical and experimental *M*_*n*_ shifts up to 40% BzMA recovery (Table S3). Additionally, retention of the RAFT chain-ends, which is essential for controlled depolymerization, was confirmed by UV SEC analysis (Figure S8). TEM and SAXS studies were also employed to confirm the transformation from vesicles to worms (Table S4, Figure 2). More specifically, SAXS analysis revealed a shift in the low *q* gradient from – 2 for the original PLMA_16_ *-b-*PBzMA_72_ vesicles (Figure 2a) to – 1 for PLMA_16_ *-b-*PBzMA_48_ after generating 33% BzMA (Figure 2b, Figure S9), indicating the formation of worms.^63^ However, a subsequent worm-to-sphere transition could not be achieved in situ because longer reaction times resulted in the appearance of vinyl signals assigned to LMA monomer in addition to those corresponding to BzMA monomer (Figure S10). Furthermore, the use of an alternative initiator highlighted the trade-off between reaction kinetics and controlled monomer regeneration. Using azobisisobutyronitrile (AIBN; *k*_d_ = 1.5 × 10^–3^ s^–1^ at 100 °C; t_1__/__2_ = 10 h at 65 °C in toluene^62^) led to rapid generation of BzMA monomer, albeit in a lower overall yield of 30% (vs 40% with ABCN, Figure S11), and NMR vinyl signals assigned to LMA were observed earlier during this depolymerization experiment. This reflects the faster rate of thermal decomposition for AIBN at 100 °C and chain-end activation. Importantly, the vesicle-to-worm transformation remained unaffected within the window for controlled release.

**Figure 2:**
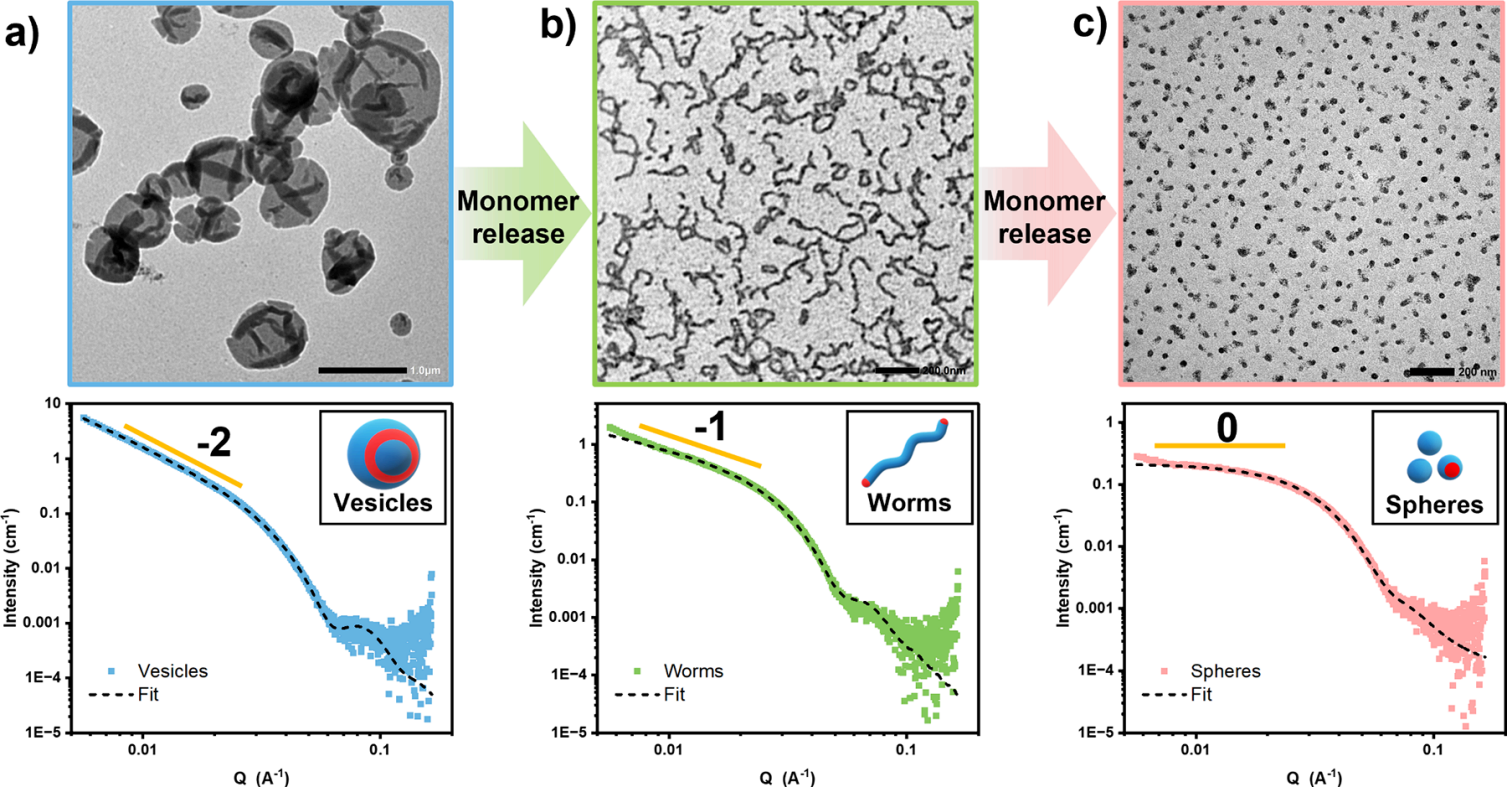
Representative TEM images (top) and SAXS patterns (bottom) recorded for (a) PLMA_16_ *-b-*PBzMA_72_ vesicles (TEM image scale bar = 1.0 μm), (b) PLMA_16_ *-b-*PBzMA_48_ worms (TEM image scale bar = 200 nm), and (c) PLMA_16_ *-b-*PBzMA_36_ spheres (TEM image scale bar = 200 nm) produced via DIMT.

To evaluate whether this system also allow access to spher-es, the initial diblock copolymer composition was adjusted to PLMA_16_ *-b-*PBzMA_56_, which led to an initial mixture of vesicles and worms (Figure S12). Following successful depolymerization, worms could be detected for a series of PLMA_16_ *-b-*PBzMA_53–43_ nanoparticles corresponding to BzMA yields of up to 23% (Table S5). Pleasingly, small spheres could also be obtained after additional depolymerization (which corresponds to PLMA_16_ *-b-*PBzMA_39–35_ nanoparticles). These findings were supported by both TEM and SAXS analyses. SAXS indicated a low *q* gradient of zero, which corresponds to the formation of PLMA_16_ *-b-*PBzMA_36_ spheres (Figure 2c). Notably, the overlapping region from both kinetic experiments (DP 56 to 43) yielded almost identical morphologies (Figure S13a), illustrating that comparable morphologies can be produced via depolymerization regardless of the initial PBzMA block DP, further highlighting the importance of the DP of the core-forming block for governing self-assembly (when using a PLMA_16_ stabilizer block). The combined TEM images obtained from both experiments provided a detailed reconstruction of the depolymerization-induced morphological evolution when using this soluble precursor (Figure S13b).

Guided by these preliminary experiments, we sought to avoid the initial mixed vesicles/worm morphology exhibited by PLMA_16_ *-b-*PBzMA_56_ nanoparticles and instead achieve the complete transformation of vesicles into spheres via worms. Accordingly, PLMA_15_ *-b-*PBzMA_63_ nanoparticles were synthesized (Figure S14). After generating just 6% BzMA, the morphology switched from vesicles to a mixed vesicle/worm phase (PLMA_15_ *-b-*PBzMA_59_), while pure worms were obtained at 19% BzMA generation, or PLMA_15_ *-b-*PBzMA_51_ (Table S6, Figure S15). Continued PBzMA depolymerization up to 32% retained the worm morphology for PLMA_15_ *-b-*PBzMA_43_. Gratifyingly, a pure sphere phase was observed in the latter stages of depolymerization (PLMA_15_ *-b-*PBzMA_39–36_) after around 40% of BzMA recovery. To the best of our knowledge, this is the first example of a complete depolymerization-induced morphological transformation from vesicles to worms to spheres for a single diblock copolymer.

To construct a (pseudo)­phase diagram, a series of new PLMA*-b-*PBzMA diblock copolymers were also prepared. For example, PLMA_14_ *-b-*PBzMA_58_ vesicles (Figure S16) exhibited a depolymerization-induced morphological transition to PLMA_14_ *-b-*PBzMA_48_ worms and subsequently PLMA_14_ *-b-*PBzMA_38_ spheres (Table S7, Figure S17). Furthermore, depolymerization of PLMA_16_ *-b-*PBzMA_83_, PLMA_15_ *-b-*PBzMA_76_ and PLMA_14_ *-b-*PBzMA_76_ provided additional information regarding the vesicle phase. Vesicles were observed for PBzMA DP 83–71 for PLMA_16_-*b*-PBzMA_83_ (Table S8), DP = 76–65 for PLMA_15_-*b*-PBzMA_76_ (Table S9), and DP 76–56 for PLMA_14_-*b*-PBzMA_76_ (Table S10), respectively. As shown in Figure 3, a clear correlation was established between higher-order morphologies and higher core-forming block/stabilizer block ratios (PBzMA/PLMA). Notably, using a longer stabilizer block (PLMA_16_ *)* expanded the accessible morphological window. Specifically, PLMA_16_ formulations formed pure vesicles above a block ratio of 4.2, transitioning to mixed vesicles/worms (4.2–3.4), pure worms (3.4–2.6), and spheres (<2.6). Conversely, limiting the steric stabilizer DP (PLMA_14_) constrained this range, shifting the boundaries for mixed phases and worms to PBzMA/PLMA ratios of 3.9–3.6 and 3.6–2.9, respectively. Notably, in systems with longer stabilizer blocks (PLMA_22_-*b*-PBzMA_63_) where exclusively spherical morphologies are obtained even when targeting a high DP for the PBzMA block (Figure S18), DIMT led to a continuous reduction in particle diameter, rather than an evolution in copolymer morphology (Figure S19, Table S11). It is important to note that the term ‘pseudophase diagram’ is used because PISA may lead to kinetically trapped morphologies in some cases, rather than thermodynamically preferred equilibrium structures.

**Figure 3:**
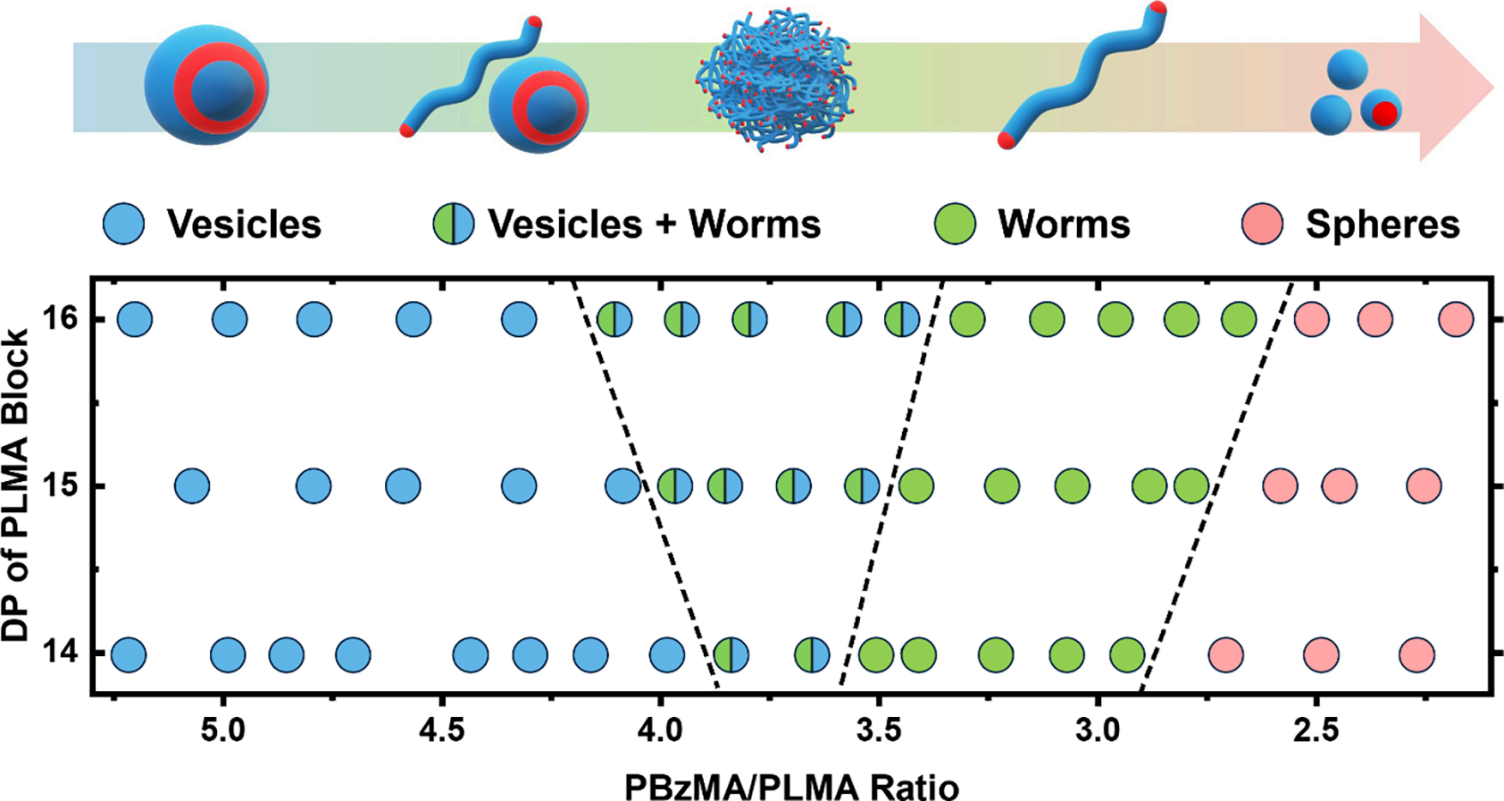
(Pseudo)­phase diagram constructed from multiple DIMT experiments for the PLMA*-b-*PBzMA system.

Remarkably, the DIMT-derived (pseudo)­phase diagram closely mirrors that reported for PISA syntheses, despite significant differences in experimental conditions (i.e., depolymerization versus polymerization, reaction temperature (80 °C vs 100 °C), and solids content (20 wt % vs 0.7 wt %, Figure S20). These results again suggest that the mean DP of each block governs the copolymer morphology in PISA syntheses because the opposite sequence of morphological transitions occurred in DIMT despite its significantly lower copolymer concentration. This is because PISA involves an evolution in copolymer morphology via an associative pathway (i.e., via an increase in the mean aggregation number), whereas DIMT involves a purely dissociative pathway. The former pathway is known to be concentration-dependent, whereas the latter pathway is expected to be independent of the copolymer concentration. Therefore, the development of DIMT enables future comprehensive studies through a dissociative and concentration-independent pathway. Furthermore, we demonstrated that both depolymerization and (partial) repolymerization can be achieved for the PISA system. TEM analysis confirmed the complete regeneration of the original mixed phase from the intermediate worm phase (Figure S21), accompanied by a change in visual appearance of the reaction mixture from turbid to transparent to turbid.

To investigate whether the BzMA monomer generated during depolymerization was swelling the nanoparticle cores and therefore was responsible for the observed morphological transformation rather than the DP reduction of the second block,^64^ nanoparticles were isolated by centrifugation and subsequently characterized by ^1^H NMR spectroscopy and TEM (Figure S22). The results showed that the majority of the monomer was dissolved in the solvent with only a very low concentration present within the nanoparticle core. Therefore, monomer-induced core swelling is unlikely to be the main reason accounting for the morphological transitions observed during depolymerization. This contrasts with our previous work, which showed that adding monomer to PISA nanoparticles could alter their morphology by swelling the nanoparticle cores at room temperature.^64^ TEM images recorded for nanoparticles obtained from the depolymerization solution were compared with those obtained for nanoparticles isolated by centrifugation, where the monomer was mostly removed. In both cases, identical worm-like morphologies were observed, suggesting that the morphological transitions resulted from the gradual reduction in molecular weight, rather than from monomer-swollen nanoparticle cores. However, this control experiment does not rule out the possibility that BzMA generation could lead to monomer-swollen PBzMA cores at the relatively high reaction temperature of 100 °C,^63^ highlighting the need for further studies on such formulations.

It has been reported that PLMA*-b-*PBzMA worms can form gels, which undergo a reversible transformation to form spherical micelles upon heating and revert to worms upon cooling.^61,65^ The initial worm-to-sphere transition is attributed to surface plasticization of the insoluble core-forming block, leading to a reversible change in its effective volume fraction.^61^ We hypothesized that applying DIMT to reduce the molecular weight of the core-forming block would produce kinetically trapped spherical nanoparticles that are unable to reform the original highly anisotropic worms, thereby preventing regelation. To test this hypothesis and assess the potential of DIMT to induce an irreversible degelation via a worm-to-sphere transition, a tube inversion test was conducted. Treatment of a 12 wt % PLMA_16_ *-b-*PBzMA_48_ gel with 0.2 equiv of ABCN initiator at 120 °C resulted in the expected irreversible degelation (Figure S23), accompanied by 17% monomer regeneration and a reduction in the mean DP of the core-forming PBzMA block to 40. In contrast, the control experiment conducted in the absence of any ABCN initiator exhibited the expected reversible regelation behavior after a thermal cycle. The resulting spheres generated at elevated temperature remained dynamic and readily reformed worms via multiple 1D fusion events on cooling, reconstituting the original worm gel.^61^ These findings establish depolymerization as a powerful strategy for irreversibly modulating the gelation behavior of diblock copolymer nanoparticles through precise tuning of the core block volume fraction (or DP).

Finally, it is worth noting that, although the core-forming block is currently limited to methacrylates due to thermodynamic constraints, the soluble stabilizer block is not subject to such restrictions. Chemically inert polybutadiene or poly­(ethylene glycol)^66–68^ stabilizers can be employed, as they do not participate in depolymerization. While such hybrid systems require further investigation, this suggests that the concept of reverse PISA could be extended to other vinyl polymer classes. It is also anticipated that this strategy could be extended to various nonpolar solvents and PISA systems, while it can also be employed to reduce the size of kinetically trapped spherical nanoparticles.

## Conclusions

In conclusion, we report the first example of depolymerization-induced morphological transformation for vinyl polymers, enabling complete morphological transitions from vesicles to worms to spheres. Well-controlled monomer generation originating exclusively from the core-forming block is achieved by selecting a relatively mild temperature (100 °C) and adding a suitable radical initiator. This strategy allows sequential depolymerization of the insoluble structure-directing PBzMA block up to 40–45% conversion, resulting in a gradual reduction in the relative volume fraction of this component. The controlled monomer release was confirmed by a reduction in molecular weight, which closely matched theoretical predictions. The resulting stepwise morphological vesicle-to-worm and worm-to-sphere transitions were validated by TEM and SAXS studies, demonstrating modular dual control that directly links precise molecular weight reduction to a corresponding tunable morphological evolution. This feature uniquely distinguishes the present approach from previously reported depolymerization systems. Moreover, multiple depolymerization experiments facilitated the construction of a (pseudo)­phase diagram. The close agreement between the copolymer morphologies obtained via PISA and DIMT confirms the critical role of the block volume fraction (or DP) in determining the copolymer morphology, despite the significantly lower copolymer concentrations employed in DIMT. Finally, DIMT was utilized to achieve irreversible degelation, whereby a block copolymer worm gel underwent degelation via a worm-to-sphere transition, resulting in a low-viscosity fluid as confirmed by visual inspection (tube inversion test). This behavior highlights the potential of DIMT not only for the efficient chemical recycling of methacrylic block copolymers, but also for broader applications in materials processing and fundamental studies of self-assembly mechanisms.

## Supplementary Material



## Notes

General information, experimental procedures, ^1^H NMR spectra, and SEC traces (PDF)

## Abbreviations

DP: degree of polymerization
SEC: size exclusion chromatography
SAXS: small-angle X-ray scattering
TEM: transmission electron microscopy
