# Supplementary material for: Depolymerization-Induced Morphological Transformation
Source: J Am Chem Soc. 2026 Jan 26;148(5):5400–8. doi: 10.1021/jacs.5c18937 (PMC12903855; doi:10.1021/jacs.5c18937)
Supplement: Supplementary file 1 [file ja5c18937_si_001.pdf]

Depolymerization-Induced Morphological Transformation (DIMT)

Nethmi De Alwis Watuthanthrige,[1] Victoria Lohmann,[1] Viviane Lutz-Bueno,[2] Nghia P. Truong,[1] Steven P. Armes,[3]* Athina Anastasaki[1]*

[1]Laboratory for Sustainable Polymers, Department of Materials, ETH Zürich, Vladimir-Prelog-Weg 5, 8093 Zürich, Switzerland

[2]PSI Center for Neutron and Muon Sciences, 5232 Villigen PSI, Switzerland

[3]School of Mathematical and Physical Sciences, Dainton Building, University of Sheffield, Sheffield, South Yorkshire S3 7HF, U.K

Contents

1. Methods and Materials 4
  1.1. Materials 4
  1.2. Nuclear Magnetic Resonance (NMR) spectroscopy 4
  1.3. Size-exclusion Chromatography (SEC) 4
  1.4 Dynamic Light Scattering (DLS) 5
  1.5 Transmission Electron Microscopy (TEM) 5
    1.5.1 Sample Preparation for PISA 5
    1.5.2 Sample Preparation for DIMT 5
    1.5.3 Staining and Imaging 5
  1.6 Small Angle X-Ray Scattering (SAXS) 6
2 Experimental 6
  2.1 Synthesis Procedures of Macro-CTAs 6
    2.1.1 Synthesis of Poly(Lauryl Methacrylate)$_{16}$ (PLMA$_{16}$) 6
    2.1.2 Synthesis of PLMA$_{15}$ 7
    2.1.3 Synthesis of PLMA$_{14}$ 7
    2.1.4 Synthesis of PLMA$_{22}$ 7
  2.2 Synthesis of Poly(Lauryl Methacrylate)-*b*-Poly(Benzyl Methacrylate) (PLMA-*b*-PBzMA) Diblock Copolymer Particles 7
    2.2.1 Synthesis of PLMA$_{16}$-*b*-PBzMA$_{72}$ 7
    2.2.2 Synthesis of PLMA$_{16}$-*b*-PBzMA$_{56}$ 8
    2.2.3 Synthesis of PLMA$_{15}$-*b*-PBzMA$_{63}$ 8
    2.2.4 Synthesis of PLMA$_{14}$-*b*-PBzMA$_{58}$ 8
    2.2.5 Synthesis of PLMA$_{16}$-*b*-PBzMA$_{83}$ 9
    2.2.6 Synthesis of PLMA$_{15}$-*b*-PBzMA$_{76}$ 9
    2.2.7 Synthesis of PLMA$_{14}$-*b*-PBzMA$_{76}$ 9
    2.2.8 Synthesis of PLMA$_{22}$-*b*-PBzMA$_{63}$ 9
  2.3 Typical Depolymerization Reaction of PLMA-*b*-PBzMA Diblock Copolymer Particles 10
    2.3.1 Depolymerization of PLMA-*b*-PBzMA (10 mL Scale) 10
    2.3.2 Depolymerization of PLMA-*b*-PBzMA (30 mL Scale) 10
    2.3.3 Depolymerization with Varying ABCN Amounts 11
    2.3.4 Depolymerization with Azobisisobutyronitrile (AIBN) 11
  2.4 Thermal Annealing Test 11
  2.5 In Situ Depolymerization-Repolymerization Cycle 11

2.6 Isolation of the Nanoparticles 12
2.7 Calculation of BzMA Monomer Regeneration from ¹H-NMR Analysis 12
2.8 Calculation of the DP of the PBzMA after Depolymerization 13
2.9 Tube Inversion Test 13
2.9.1 Synthesis of $PLMA_{16}$-*b*-$PBzMA_{48}$ 13
2.9.2 Tube Inversion Test (Heating vs Depolymerization) 14
3 Additional Figures and Tables 15
3.1 Morphological Transformation of Block Copolymers with $PLMA_{16}$ Stabilizer Block 17
3.1.1 Vesicles to Worms Transformation using $PLMA_{16}$-*b*-$PBzMA_{72}$ 17
3.1.2 Vesicles + Worms to Spheres Transformation using $PLMA_{16}$-*b*-$PBzMA_{56}$ 22
3.2 Morphological Transformation of Block Copolymers with $PLMA_{15}$ Stabilizer Block 24
3.3 Morphological Transformation of Block Copolymers with $PLMA_{14}$ Stabilizer Block 25
3.4 Additional Kinetic Experiments to Complete the Phase Diagram 26
3.5 Depolymerization of PLMA-b-PBzMA System with Longer Stabilizer Block ($PLMA_{22}$) 27
3.6 Polymerization vs Depolymerization 29
3.7 Demonstration of Reversibility via an In Situ Depolymerization–Repolymerization Cycle ... 29
3.8 Isolation of Nanoparticles 30
3.9 Tube Inversion Test 30
4 References 30

1. Methods and Materials

1.1. Materials

All materials were purchased from either Sigma Aldrich or Fisher Scientific unless otherwise stated. Monomers were purified by passing through basic alumina before use.

1.2. Nuclear Magnetic Resonance (NMR) spectroscopy

^{1}H-NMR spectra were recorded on a Bruker Avance-300 spectrometer using $CDCl_3$ as the NMR solvent. Spectra were referenced to the residual chloroform peak.

1.3. Size-exclusion Chromatography (SEC)

SEC was measured on Shimadzu equipment comprising a CBM-20A system controller, LC-20AD pump, SIL-20A automatic injector, 10.0 µm bead-size guard column (50 x 7.5 mm) followed by three KF-805L columns (300 x 8 mm, bead size: 10 µm, pore size maximum: 5000 Å), SPD-20A ultraviolet (UV) detector, and an RID-20A differential refractive index (RI) detector. The column temperature was maintained at 40 °C using a CTO-20A oven. The flow rate was set to 1 mL/min and with *N,N*-dimethylacetamide (DMAc, Sigma-Aldrich, HPLC grade, with 0.03 w/v LiBr) as the eluent. Molecular weights were determined relative to poly(methyl methacrylate) standards with molecular weights ranging from 5,000 to 1.5 x 10^6 g/mol (EasiVial PMMA, Agilent Technologies). All SEC samples were prepared by dissolution in DMAc and passed through a 0.45 µm PTFE syringe filter prior to analysis.

Poly(lauryl methacrylate) (PLMA) analysis was performed using a SEC LF-804 column with tetrahydrofuran as the eluent at 35 °C on an Agilent 1260 Infinity II system (THF SEC) with a flow rate of 1.0 mL/min. SEC samples were prepared by dissolving the sample in THF and passing through a 0.45 µm PTFE syringe filter prior to analysis.

1.4 Dynamic Light Scattering (DLS)

Measurements were carried out using a Malvern Zetasizer Nano Series ZS, employing a backscatter detection system at 173° angle and a standard laser (4 mW, 633 nm). The sample refractive index (RI) was set at 1.57 for poly(benzyl methacrylate) (PBzMA). The dispersant viscosity and RI were set to 0.89 Ns m^{-2} and 1.33, respectively.

Samples from depolymerization mixtures were analyzed directly without further dilution (0.7 wt% solid content).

1.5 Transmission Electron Microscopy (TEM)

1.5.1 Sample Preparation for PISA

TEM samples of the polymerization-induced self-assembly (PISA) mixtures were prepared by diluting approximately 10 mg of the reaction mixture (20 wt%) with 1 mL of dodecane to obtain a 0.2 wt% polymer solution. Then, 1 µL of the diluted solution was deposited onto 300-mesh carbon-coated Formvar grids using the drop-casting method and allowed to dry overnight at room temperature.

1.5.2 Sample Preparation for DIMT

TEM samples of the depolymerization-induced morphological transformation (DIMT) mixtures were prepared by diluting approximately 10 µL of the reaction mixture (0.7 wt%) with 30 µL of dodecane to obtain a ~0.2 wt% polymer solution. Approximately 1.5 µL of the diluted solution was then deposited onto 300-mesh carbon-coated Formvar grids using the drop-casting method and allowed to dry overnight at room temperature.

1.5.3 Staining and Imaging

The drop-casted TEM grids were exposed to ruthenium(IV) oxide vapor for 30 minutes at room temperature, as depicted in Scheme 1, by placing a Petri dish in a sealed container. This heavy-metal staining enhanced contrast by selectively staining the core-forming PBzMA block. The ruthenium(IV) oxide solution was prepared according to established protocols.[1]

Imaging was carried out using a Jeol JEM 1400 (High Voltage: 80 kV, 120 kV, Emitter: LaB_6 crystal) transmission electron microscope.

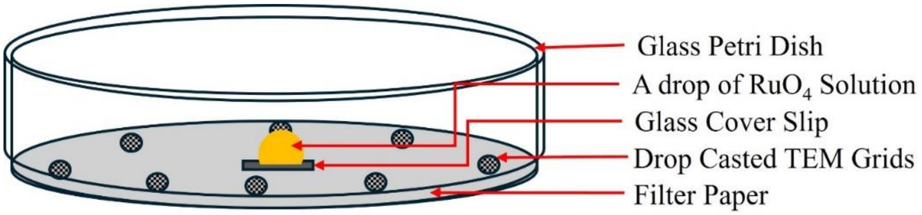

Scheme S1: Schematic illustration of the setup used for staining TEM grids with RuO_4 vapor in a glass Petri dish for 30 min.

1.6 Small Angle X-Ray Scattering (SAXS)

SAXS measurements were performed using a Xenocs Xeuss 3.0 system. This system was equipped with a Dectris EIGER2 detector and a Copper K-α source (λ = 0.154 nm). Measurements were taken in a vacuum at two detector-to-sample distances of 0.3 m and 1.65 m. The scattering intensity was recorded as a function of the scattering vector q (in reciprocal units relative to the wavelength). Samples were measured in quartz capillaries with 1 mm thickness. The scattering signal of a capillary filled with water was subtracted from the measurements as a background correction, after normalizing based on sample X-ray transmission and measurement time.

2 Experimental

2.1 Synthesis Procedures of Macro-CTAs

2.1.1 Synthesis of Poly(Lauryl Methacrylate)$_{16}$ (PLMA$_{16}$)

The polymerization protocols for PLMA were adapted from previously published literature.[1]

In a 25 mL round-bottom flask equipped with a magnetic stir bar, 261 mg of 2-cyanopropan-2-yl benzodithioate (1.18 mmol, 1 equivalent), 38.7 mg of AIBN (236 µmol, 0.2 equivalents), and 3.00 g of lauryl methacrylate (LMA) (11.8 mmol, 10 equivalents) were dissolved in 4.95 g of toluene. The flask was sealed with a septum and deoxygenated by nitrogen gas sparging for 15 minutes.

Polymerization was carried out in an oil bath at 80 °C with stirring at 400 rpm. Periodic samples were collected under a nitrogen blanket for ^{1}H NMR analysis to determine the conversion of the LMA. Polymerization was terminated at 90–96% monomer conversion (approximately 4 h) by removing the flask from the oil bath and unsealing the septum.

The polymer was then precipitated into excess methanol three times and dried under vacuum. It was analyzed by SEC in THF.

2.1.2 Synthesis of $PLMA_{15}$

The synthesis and purification of $PLMA_{15}$ was performed using the same molar equivalents and identical procedure as for the $PLMA_{16}$ polymerization. The reaction was stopped at approximately 79% conversion (around 2 h and 30 minutes), resulting in the formation of $PLMA_{15}$.

2.1.3 Synthesis of $PLMA_{14}$

The synthesis and purification of $PLMA_{14}$ was performed using the identical procedure as for the $PLMA_{16}$ polymerization, with adjusted reactant equivalents: 2-cyanopropan-2-yl benzodithioate (326.3 mg, 1.5 mmol, 1 equivalent), 2,2′-Azobis(2-methylpropionitrile) (AIBN) (48.4 mg, 295 µmol, 0.2 equivalents), and LMA (3.00 g, 11.8 mmol, 8 equivalents), dissolved in 5.06 g of toluene.

The reaction was stopped at approximately 90-94% conversion (around 3.30 h), resulting in the formation of $PLMA_{14}$.

2.1.4 Synthesis of $PLMA_{22}$

The synthesis and purification of $PLMA_{22}$ was performed using the identical procedure as for the $PLMA_{16}$ polymerization, with adjusted reactant equivalents: 2-cyanopropan-2-yl benzodithioate (174 mg, 0.79 mmol, 1 equivalent), 2,2′-Azobis(2-methylpropionitrile) (AIBN) (25.8 mg, 157 µmol, 0.2 equivalents), and LMA (3.00 g, 11.8 mmol, 15 equivalents), dissolved in 4.8 g of toluene.

The reaction was stopped approximately 90-94% conversion (around 3.30 h), resulting in the formation of $PLMA_{22}$.

2.2 Synthesis of Poly(Lauryl Methacrylate)-*b*-Poly(Benzyl Methacrylate) (PLMA-*b*-PBzMA) Diblock Copolymer Particles

2.2.1 Synthesis of $PLMA_{16}$-*b*-$PBzMA_{72}$

A typical synthesis of $PLMA_{16}$-*b*-$PBzMA_{72}$ via RAFT non-aqueous dispersion polymerization was conducted as previously reported.[1] In a 10 mL round-bottom flask equipped with a stirrer bar, benzyl methacrylate (BzMA, 590.2 mg, 3.45 mmol, 74 equivalents), AIBN (1.53 mg, 9.32 µmol, 0.2 equivalents), and $PLMA_{16}$ macro-CTA (200 mg, 46.6 µmol, 1 equivalent) were dissolved in

3.17 g of *n*-dodecane (20 wt% solid content). The flask was sealed with a septum and deoxygenated by nitrogen gas sparging for 15 minutes.

Polymerization was conducted in an oil bath at 80 °C with stirring at 400 rpm. Periodic samples were collected under a nitrogen blanket for ^{1}H-NMR analysis to determine the conversion of BzMA. The polymerization was terminated after reaching >95% monomer conversion (after approximately 4 h) by cooling to room temperature and opening the flask to air.

To synthesize polymers with varying DPs of BzMA, the mean DP of the PBzMA block was systematically adjusted by altering the amount of added BzMA monomer while maintaining identical reaction conditions.

2.2.2 Synthesis of PLMA$_{16}$-*b*-PBzMA$_{56}$

The synthesis of PLMA$_{16}$-*b*-PBzMA$_{56}$ was performed under identical conditions as the synthesis of PLMA$_{16}$-*b*-PBzMA$_{72}$ with adjusted reactant equivalents: BzMA (357 mg, 2.03 mmol, 58 equivalents), AIBN initiator (1.15 mg, 7.0 µmol, 0.2 equivalents), and PLMA$_{16}$ macro-CTA (150 mg, 35 µmol, 1 equivalent) and 2.03 g of *n*-dodecane (20 wt% solid content). The polymerization was terminated after reaching >95% monomer conversion (approximately 5.5 h) by cooling to room temperature and opening the flask to air.

2.2.3 Synthesis of PLMA$_{15}$-*b*-PBzMA$_{63}$

The synthesis of PLMA$_{15}$-*b*-PBzMA$_{63}$ was performed under identical conditions as the synthesis of PLMA$_{16}$-*b*-PBzMA$_{72}$ with adjusted reactant equivalents: BzMA (426 mg, 2.42 mmol, 65 equivalents), AIBN (1.22 mg, 7.4 µmol, 0.2 equivalents), PLMA$_{15}$ macro-CTA (150 mg, 37 µmol, 1 equivalent) and 2.31 g of *n*-dodecane (20 wt% solid content). The polymerization was terminated after reaching >95% monomer conversion (approximately 5.5 h) by cooling to room temperature and opening the flask to air.

2.2.4 Synthesis of PLMA$_{14}$-*b*-PBzMA$_{58}$

The synthesis of PLMA$_{14}$-*b*-PBzMA$_{58}$ was performed under identical conditions as the synthesis of PLMA$_{16}$-*b*-PBzMA$_{72}$ with adjusted reactant equivalents: BzMA (419 mg, 2.38 mmol, 60 equivalents), AIBN (1.3 mg, 7.9 µmol, 0.2 equivalents), PLMA$_{14}$ macro-CTA (150 mg, 40 µmol, 1 equivalent) and 2.28 g of *n*-dodecane (20 wt% solid content). The polymerization was terminated

after reaching >95% monomer conversion (approximately 5 h) by cooling to room temperature and opening the flask to air.

2.2.5 Synthesis of $PLMA_{16}$-*b*-$PBzMA_{83}$

The synthesis of $PLMA_{16}$-*b*-$PBzMA_{83}$ was performed under identical conditions as the synthesis of $PLMA_{16}$-*b*-$PBzMA_{72}$ with adjusted reactant equivalents: BzMA (523 mg, 2.97 mmol, 85 equivalents), AIBN (1.2 mg, 7 µmol, 0.2 equivalents), $PLMA_{16}$ macro-CTA (150 mg, 35 µmol, 1 equivalent) and 2.70 g of *n*-dodecane (20 wt% solid content). The polymerization was terminated after reaching >95% monomer conversion (approximately 4 h) by cooling to room temperature and opening the flask to air.

2.2.6 Synthesis of $PLMA_{15}$-*b*-$PBzMA_{76}$

The synthesis of $PLMA_{15}$-*b*-$PBzMA_{76}$ was performed under identical conditions as the synthesis of $PLMA_{16}$-*b*-$PBzMA_{72}$ with adjusted reactant equivalents: BzMA (510 mg, 2.9 mmol, 78 equivalents), AIBN (1.2 mg, 7.4 µmol, 0.2 equivalents), $PLMA_{15}$ macro-CTA (150 mg, 37 µmol, 1 equivalent) and 2.65 g of *n*-dodecane (20 wt% solid content). The polymerization was terminated after reaching >95% monomer conversion (approximately 4 h) by cooling to room temperature and opening the flask to air.

2.2.7 Synthesis of $PLMA_{14}$-*b*-$PBzMA_{76}$

The synthesis of $PLMA_{14}$-*b*-$PBzMA_{76}$ was performed under identical conditions as the synthesis of $PLMA_{16}$-*b*-$PBzMA_{72}$ with adjusted reactant equivalents: BzMA (545 mg, 3.1 mmol, 78 equivalents), AIBN (1.3 mg, 7.9 µmol, 0.2 equivalents), $PLMA_{14}$ macro-CTA (150 mg, 40 µmol, 1 equivalent) and 2.79 g of *n*-dodecane (20 wt% solid content). The polymerization was terminated after reaching >95% monomer conversion (approximately 4 h) by cooling to room temperature and opening the flask to air.

2.2.8 Synthesis of $PLMA_{22}$-*b*-$PBzMA_{63}$

The synthesis of $PLMA_{22}$-*b*-$PBzMA_{63}$ was performed under identical conditions as the synthesis of $PLMA_{16}$-*b*-$PBzMA_{72}$ with adjusted reactant equivalents: BzMA (295 mg, 1.6 mmol, 65 equivalents), AIBN (1.3 mg, 5.2 µmol, 0.2 equivalents), $PLMA_{22}$ macro-CTA (150 mg, 26 µmol, 1 equivalent) and 1.79 g of *n*-dodecane (20 wt% solid content). The polymerization was terminated after reaching >95% monomer conversion (approximately 5 h) by cooling to room temperature and opening the flask to air.

2.3 Typical Depolymerization Reaction of PLMA-*b*-PBzMA Diblock Copolymer Particles

2.3.1 Depolymerization of PLMA-*b*-PBzMA (10 mL Scale)

In a 20 mL Schlenk flask equipped with a stirrer bar, 241 mg of the PISA polymerization mixture (20 wt% $PLMA_{16}$-*b*-$PBzMA_{72}$ in *n*-dodecane; $PLMA_{16}$-*b*-$PBzMA_{72}$: 48 mg, 0.0028 mmol, 1 equivalent) were dissolved in 10 mL of dodecane and stirred thoroughly until no visible polymer chunks remained. The resulting polymer solution had a repeat unit concentration of 25 mM (0.7 wt% solid content). A stock solution of ABCN was prepared in toluene (27.3 mg/mL). 15.2 µL of the stock solution (ABCN: 0.42 mg, 0.0017 mmol, 0.6 equivalents) were added. To minimize any impact on nanoparticle morphology, the added toluene volume was kept below 0.2% of the total reaction volume.

The flask was sealed with a septum and deoxygenated by sparging with nitrogen gas for 15 minutes. The depolymerization reaction was conducted in an oil bath at 100 °C, with stirring at 400 rpm. Periodic samples were collected (200 µL) under a nitrogen blanket for ^{1}H-NMR, TEM, and SEC Analysis.

2.3.2 Depolymerization of PLMA-*b*-PBzMA (30 mL Scale)

Scale-up reactions were carried out in a 50 mL round-bottom flask to obtain full characterization of the kinetics (TEM, DLS, SEC and NMR Analysis) following the identical procedure described above with adjusted reactant amounts: 724 mg of the PISA polymerization mixture (20 wt% $PLMA_{16}$-*b*-$PBzMA_{72}$ in *n*-dodecane; $PLMA_{16}$-*b*-$PBzMA_{72}$: 145 mg, 0.00852mmol, 1 equivalent) were mixed with 30 mL of *n*-dodecane (25 mM RUC, 0.7 wt% solid content), and ABCN (0.42 mg, 0.00511 mmol, 0.6 equivalents). The ABCN was introduced using a stock solution in toluene (84 µL from a 5 mg/mL stock).

1 mL aliquots were collected at various time points and analyzed using multiple techniques: ^{1}H-NMR (90 µL aliquot diluted with 550 µL of $CDCl_3$), TEM (10 µL aliquot diluted with 30 µL of dodecane), SEC Analysis (100 µL aliquot, dried by air blowing to remove dodecane, then diluted with 1.5 mL of DMAc) and DLS Analysis (800 µL aliquot used without further dilution).

2.3.3 Depolymerization with Varying ABCN Amounts

Kinetic reactions were performed following the 2.3.1 procedure with varying ABCN equivalents (0–1.0 eq) using a stock solution of ABCN in toluene (27.3 mg/mL). The specific additions were 5.1 μL for 0.2 equivalents (0.14 mg, 0.57 μmol), 10.1 μL for 0.4 equivalents (0.28 mg, 1.13 μmol), 25.3 μL for 1.0 equivalents (0.69 mg, 2.83 μmol), while no ABCN was added for the control reaction.

2.3.4 Depolymerization with Azobisisobutyronitrile (AIBN)

Kinetic reactions were performed according to the procedure described in Section 2.3.1, using 0.6 equivalents of AIBN (0.84 mg, 0.00511 mmol). The ABCN was introduced using a stock solution in toluene (84 μL from a 10 mg/mL stock).

2.4 Thermal Annealing Test

Thermal annealing of $PLMA_{16}$-*b*-$PBzMA_{72}$ was carried out in a 50 mL round-bottom flask using 724 mg of the PISA polymerization mixture (20 wt% $PLMA_{16}$-*b*-$PBzMA_{72}$ in *n*-dodecane; $PLMA_{16}$-*b*-$PBzMA_{72}$: 145 mg, 0.00852mmol, 1 equivalent) diluted in 30 mL of *n*-dodecane (25 mM RUC, 0.7 wt% solid content). The reaction mixture was heated at 100 °C for 2 hours without deoxygenation. Kinetic analysis was conducted on 1 mL aliquots via ^{1}H-NMR (90 μL aliquot diluted with 550 μL of $CDCl_3$), TEM (10 μL aliquot diluted with 30 μL of dodecane), SEC Analysis (100 μL aliquot, dried by air blowing to remove dodecane, then diluted with 1.5 mL of DMAc), and DLS Analysis (800 μL aliquot used without further dilution).

2.5 In Situ Depolymerization-Repolymerization Cycle

The depolymerization reaction was setup according to Section 2.3 using 241 mg of the PISA polymerization mixture (20 wt% $PLMA_{16}$-*b*-$PBzMA_{68}$ in n-dodecane; containing 48 mg, 0.0028 mmol, 1 equivalent of block copolymer). To this, 0.4 equivalents of ABCN (0.28 mg, 0.0011 mmol, 0.6 equivalents; added as 12.7 μL of a 22 mg/mL stock solution in toluene) were added in 10 mL of n-dodecane, and the mixture was heated at 100 °C. The reaction was quenched after 90 min by cooling the Schlenk tube in an ice bath; ^{1}H NMR analysis indicated 25% monomer regeneration.

Subsequently, 25 µL of a BzMA stock solution containing AIBN (3.7 mg/mL) was added to the mixture. This addition corresponded to 0.2 equivalents of AIBN (0.92 mg, 0.00056 mmol) and 0.17 mmol of BzMA. The mixture was degassed for 15 min and placed in an oil bath at 80 °C. The reaction was monitored via ^{1}H NMR analysis and quenched after 240 min by cooling in an ice bath. TEM analysis was performed to confirm the morphologies following both the depolymerization and repolymerization processes.

2.6 Isolation of the Nanoparticles

For nanoparticle isolation, a depolymerization experiment was performed under the reaction conditions described above. The reaction was halted upon achieving 33% BzMA regeneration, and a portion of the sample was diluted (10 µL sample + 30 µL dodecane) to prepare TEM samples for morphological analysis.

The remaining sample was centrifuged at 7000 rpm, 25 °C, for 5 minutes to separate the nanoparticles from the solution. The isolated nanoparticles were analyzed via NMR to detect any residual monomers and were further diluted (~0.2 wt% solid in dodecane) to prepare additional TEM samples.

2.7 Calculation of BzMA Monomer Regeneration from ^{1}H-NMR Analysis

Method 1: The 5.2 -4.8 ppm region (sum of benzylic protons of the monomer (BzMA) and polymer (PBzMA)) was integrated and normalized to 2, and then vinyl peaks were integrated accordingly. Monomer regeneration was directly quantified based on the integrals of the vinyl proton signals.

Method 2: The vinyl protons of BzMA (≈5.5 ppm) were set to 1.0 in the peak integration, and the benzyl protons of PBzMA (≈4.8 ppm) were then integrated accordingly (x), as shown in Figure S5a. Monomer regeneration was then calculated by the following equation

$$\text{BzMA regeneration} = \left(\frac{Integral\ of\ vinyl\ protons}{Integral\ of\ vinyl\ protons + \left(\frac{Integral\ of\ the\ benzylic\ protons\ in\ PBzMA}{2}\right)}\right) = \left(\frac{1}{1+\left(\frac{x}{2}\right)}\right) \quad \text{Equation 1}$$

2.8 Calculation of the DP of the PBzMA after Depolymerization

The DP of PBzMA after depolymerization was determined based on NMR analysis. The DP calculation was performed by quantifying the regenerated BzMA monomer and correlating it with the initial polymer composition.

$$\text{DP of the poly(BzMA)}_x = initial\ DP\ of\ poly(BzMA) \times \left(\frac{100 - BzMA\ regeneration\ \%}{100}\right) \qquad \text{Equation 2}$$

To validate this method, we performed backbone proton analysis of the polymers using high-concentration NMR measurements and with an increased number of scans (Figure S5b). The methylene protons in the lauryl methacrylate block (around 3.8 ppm) were normalized to 16 as a reference (in $PLMA_{16}$ macro-CTA), and the corresponding methylene peak of BzMA (around 4.9 ppm) was integrated accordingly. The obtained values were then compared with the DPs calculated using the monomer regeneration-based method to ensure consistency and accuracy.

Both methods yielded comparable results. However, the majority of calculations were based on monomer regeneration, due to the varying quality of the LMA methylene peak in the NMR spectra, which depends on the sample concentration.

2.9 Tube Inversion Test

2.9.1 Synthesis of $PLMA_{16}$-*b*-$PBzMA_{48}$

The synthesis of $PLMA_{16}$-*b*-$PBzMA_{48}$ was performed using the same conditions as for the $PLMA_{16}$-*b*-$PBzMA_{72}$ polymerization (described in section 2.2.1), using the following equivalents of the compounds: BzMA (296 mg, 48 equivalents, 1.68 mmol), AIBN initiator (1.15 mg, 0.2 equivalents, 7.0 µmol), and $PLMA_{16}$ macro-CTA (150 mg, 1 equivalent, 35 µmol) and 1.78 g of dodecane (20 wt% solid content).

Polymerization was terminated after reaching >98% monomer conversion (approximately 6 h) by removing the flask from the oil bath and unsealing the septum.

2.9.2 Tube Inversion Test (Heating vs Depolymerization)

A total of 0.246 g (~49 mg of polymer in 197 mg of dodecane) of the synthesized 20 wt% $PLMA_{16}$-b-$PBzMA_{48}$ PISA mixture (described in section 2.6.1) was weighed into a 2 mL vial. In a separate vial, a stock solution was prepared by dissolving 1.9 mg of ABCN in 100 µL of toluene. From this stock, 10 µL (containing 0.19 mg of ABCN, corresponding to 0.2 equivalents relative to the RAFT end group) was transferred to the polymer vial. The polymer mixture was then diluted with an additional 200 µL (150 mg) of dodecane to obtain ~12 wt% solid content, mixed thoroughly, and allowed to settle at the bottom of the vial. Once the polymer had settled, the vial was deoxygenated by nitrogen gas sparging for 15 minutes and heated at 120 °C in an oil bath for 3 h.

Images of the vials were captured at three time points: initially at 25 °C, immediately after removal from the 120 °C oil bath, and after cooling to room temperature (25 °C) after sitting for 30 minutes. NMR spectra were recorded immediately after removal from the oil bath. TEM images were obtained both before heating (at 25 °C) and after cooling to 25 °C following heating.

As a control, a tube inversion test was performed under identical conditions, except without the addition of ABCN to the mixture.

3 Additional Figures and Tables

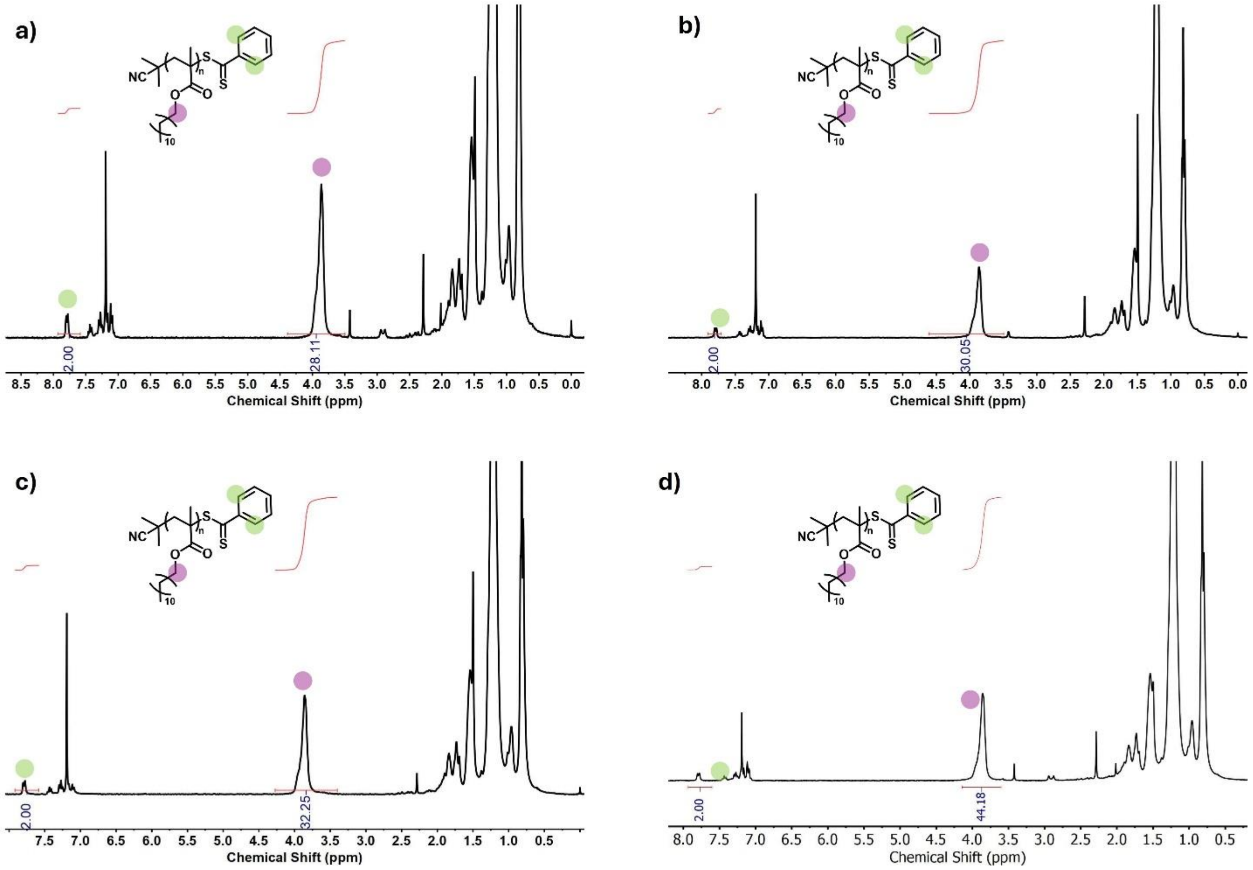

Figure S1: ^{1}H-NMR of a) DP 14, b) DP 15, c) DP 16 and d) DP22 PLMA. The exact degree of polymerization (DP) of PLMA was determined using ^{1}H-NMR spectroscopy. The aromatic protons from the dithiobenzoate end-group, located at 7.6 ppm, were integrated and normalized to a value of 2 (representing two aromatic protons). The methylene protons of the LMA repeat units appear at around 3.9 ppm. Since each LMA unit contributes two methylene protons, the DP was calculated by dividing the integration of this methylene peak by 2.

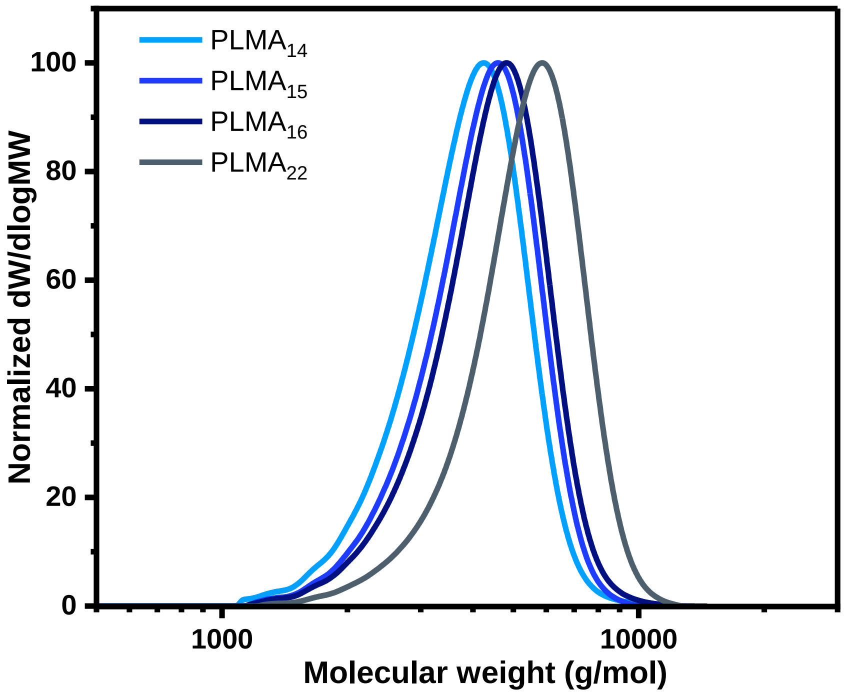

Figure S2: SEC traces of DP 14, 15 and 16 PLMA.

Table S1: Details of the polymers: DP 14, 15, 16 and 22 PLMA.

Polymer	M_n	$Đ$	livingness
$PLMA_{14}$	3600	1.11	98.6
$PLMA_{15}$	3900	1.11	98.7
$PLMA_{16}$	4100	1.11	98.4
$PLMA_{22}$	5000	1.11	98.3

[a] Calculated by the RAFT livingness equation[2]: $L = \frac{[CTA]_0}{[CTA]_0 + 2f[I_0](1-e^{-k_d t})(1-\frac{f_c}{2})}$ where $[CTA]_0$ and $[I]_0$ are the initial concentrations of chain transfer agent and initiator, respectively. The term "2" means that one molecule of initiator gives two primary radicals with a certain efficiency f (typically 0.5 for diazo initiators). The term $1 - f_c/2$ represents the number of chains produced in a radical–radical termination event with f_c the coupling factor (f_c = 1 means 100% bimolecular termination by combination; f_c = 0 means 100% bimolecular termination by disproportionation). The decomposition rate constant of the initiator k_d was 0.000032 s^{-1}.[3]

3.1 Morphological Transformation of Block Copolymers with $PLMA_{16}$ Stabilizer Block

3.1.1 Vesicles to Worms Transformation using $PLMA_{16}$-*b*-$PBzMA_{72}$

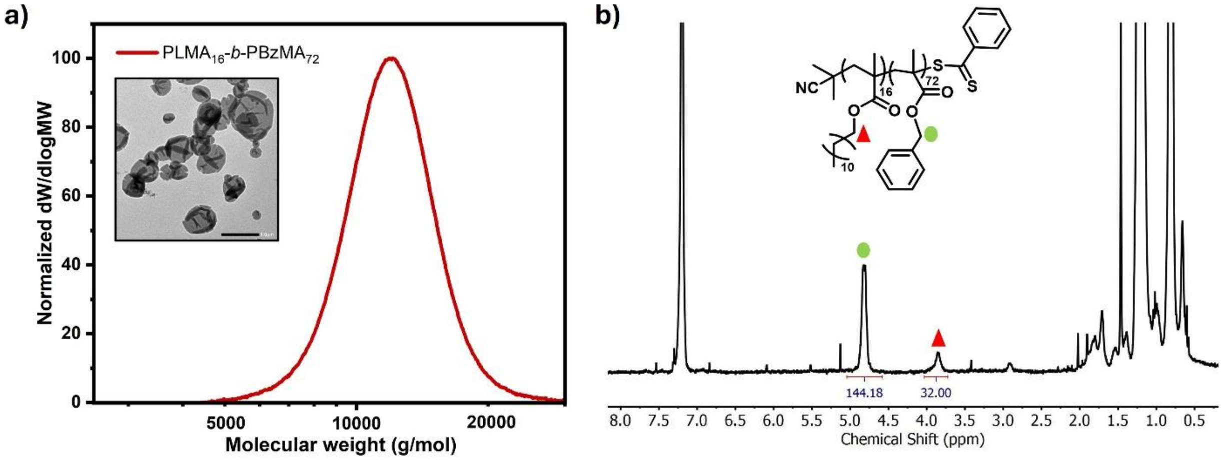

Figure S3: Characterization of $PLMA_{16}$-*b*-$PBzMA_{72}$: a) SEC trace (M_n = 11500, $Đ$ = 1.1), inset: TEM image of obtained morphology (vesicles), scale bar 1.0 µm, b) ^{1}H-NMR spectrum for $PLMA_{16}$-*b*-$PBzMA_{72}$ after 96% conversion of BzMA in 20 wt% dodecane. The exact degree of polymerization (DP) was confirmed by integrating the methylene protons of PLMA at 3.8 ppm, assigning them to 32 protons (16 repeat units × 2 protons), and then integrating the benzylic protons of PBzMA at 4.9 ppm accordingly (144 protons correspond to 72 units of BzMA, as each repeating unit contains 2 benzylic protons).

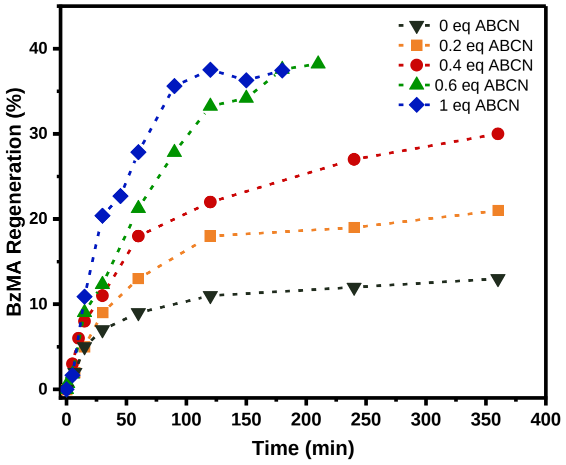

Figure S4: Effect of varying the number of equivalents of ABCN initiator on the depolymerization reaction for $PLMA_{16}$-$PBzMA_{72}$ vesicles at 100 °C, with 25 mM RUC of polymer.

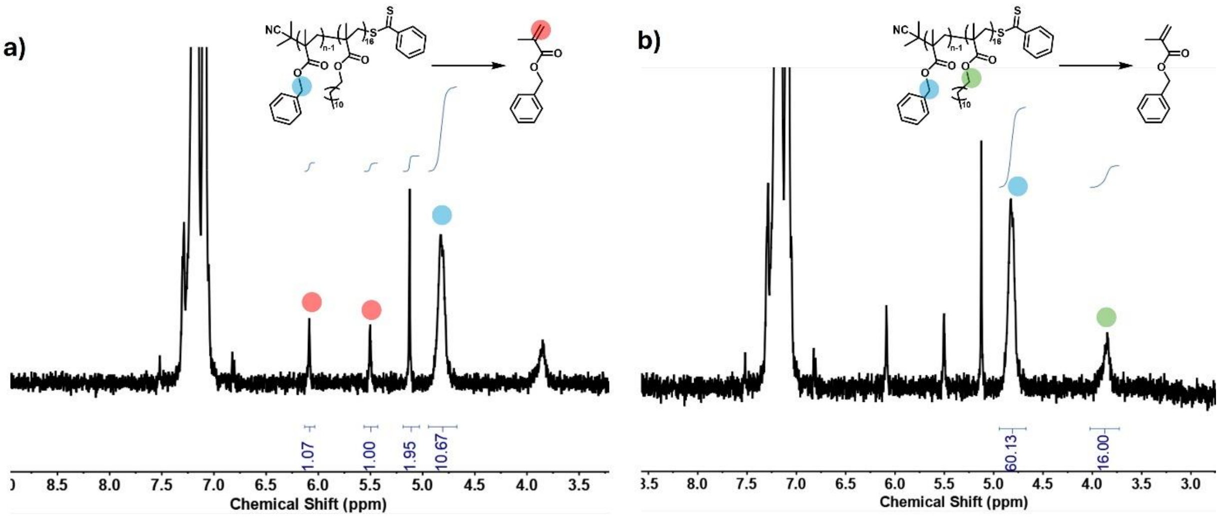

Figure S5: Methods of calculating the DPs after depolymerization of $PLMA_{16}$-*b*-$PBzMA_{72}$, a). Calculation by the extent of BzMA regeneration using equation S1 (15.2% BzMA regeneration, DP 60.7), b) calculation by polymer backbone integration (DP 60.1).

Table S2: Comparison between calculated DPs by both methods (Figure S5) for $PLMA_{16}$-*b*-$PBzMA_{72}$ block copolymer system.

Time (min)	Monomer Regeneration (%)		DP by Method 1	DP by Method 2
	BzMA	LMA		
0	0	0	72	72
5	3	0	70	70
15	8	0	66	67
30	15	0	61	60
60	27	0	53	54
90	33	0	48	47
120	40	0	42	43

Table S3: NMR and SEC analysis data from the depolymerization of $PLMA_{16}$-*b*-$PBzMA_{72}$ under the standard depolymerization conditions described in section 2.3.

Time (min)	Monomer Regeneration (%) BzMA[a]	LMA	[b]Calculated DP of $PBzMA_x$	$M_{n(exp)}$	[c]M_n shift$_{(exp)}$ (%)	[d]M_n shift$_{(th)}$ (%)
0	0	0	72	11500	0	0
1	2	0	71	11400	1	1
5	3	0	70	10800	6	2
15	8	0	66	10400	10	6
30	15	0	61	9800	15	11
45	21	0	57	9400	18	15
60	27	0	53	9100	21	19
90	33	0	48	8500	26	23

120	40	0	43	8300	28	28

[a] Calculated according to section 2.5. [b] Calculated according to section 2.6. [c] Calculated by: $M_{n(\exp)} = \frac{M_{n,0} - M_{n,t}}{M_{n,0}} \times 100\%$ [d] Calculated by: $M_{n(th,shift)} = \left(\frac{(inital\,M_{n(th)}) - (M_{n(th)} at\,time\,t)}{(inital\,M_{n(th)})}\right) \times 100\%$ with $M_{n(th)} = M_{n(th)}\ of\ PLMA_{16} - b - PBzMA_{72}$ and $M_{n(th)} at\ time\ t = M_{n(th)}\ of\ PLMA_{16} - b - PBzMA_{x}$, where x is the calculated DP of PBzMA block.

Table S4: The morphological transformation obtained through depolymerization of $PLMA_{16}$-*b*-$PBzMA_{72}$.

Time (min)	Monomer Regeneration (%)		[b]Calculated DP of PBzMA	[c]Observed Morphologies
	BzMA	LMA		
0	0	0	72	Vesicles
1	2	0	71	Vesicles
5	3	0	70	Vesicles
15	8	0	66	Vesicles+worms
30	15	0	61	Vesicles+worms
45	21	0	57	Vesicles+worms
60	27	0	53	Worms
90	33	0	48	Worms
120	40	0	43	worms

[a] Calculated according to section 2.5. [b] Calculated according to section 2.6. [c] Morphologies were observed by TEM.

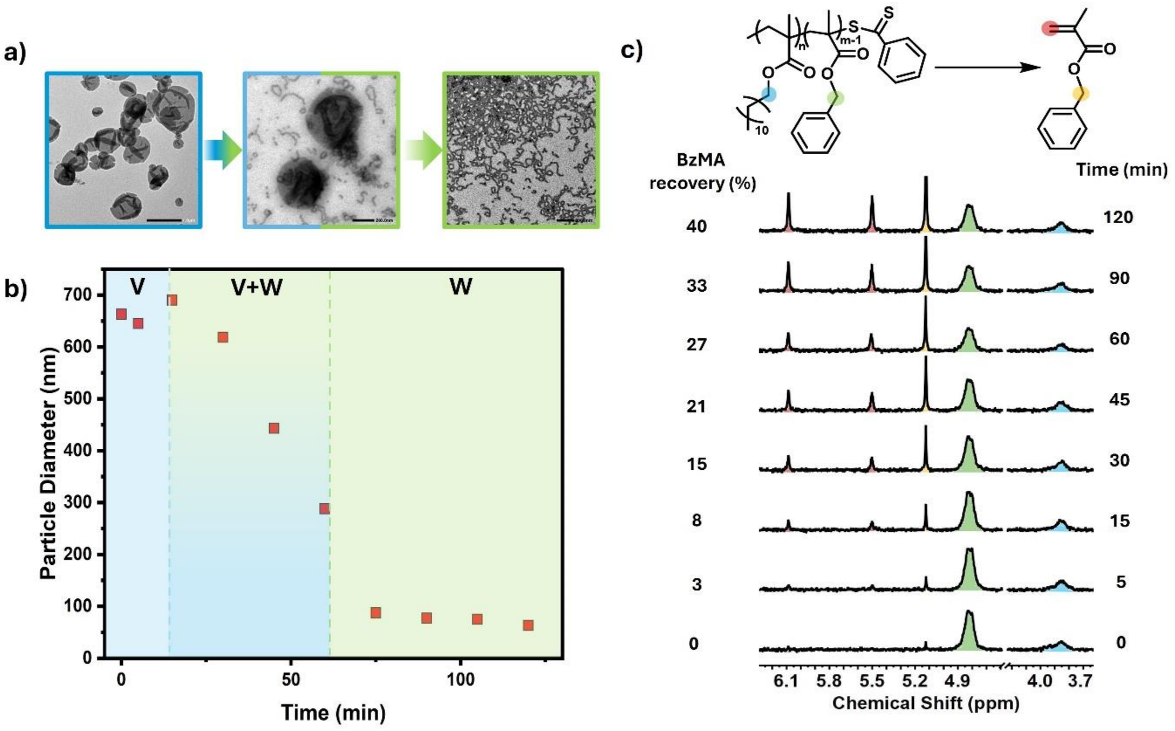

Figure S6: a) TEM images of morphological transformation from vesicles to mixed phase to worms during depolymerization of $PLMA_{16}$-b-$PBzMA_{72}$ scale bar = 1 µm (vesicles), 200 nm (Vesicles + worms), 200 nm (worms),

b) changes in the particle size upon depolymerization of $PLMA_{16}$-b-$PBzMA_{72}$ (V=vesicles, V+W=vesicles+worms, W=worms) as determined by DLS, *c)* Stacked ^{1}H-NMR traces of $PLMA_{16}$-b-$PBzMA_{72}$ depolymerization kinetics.

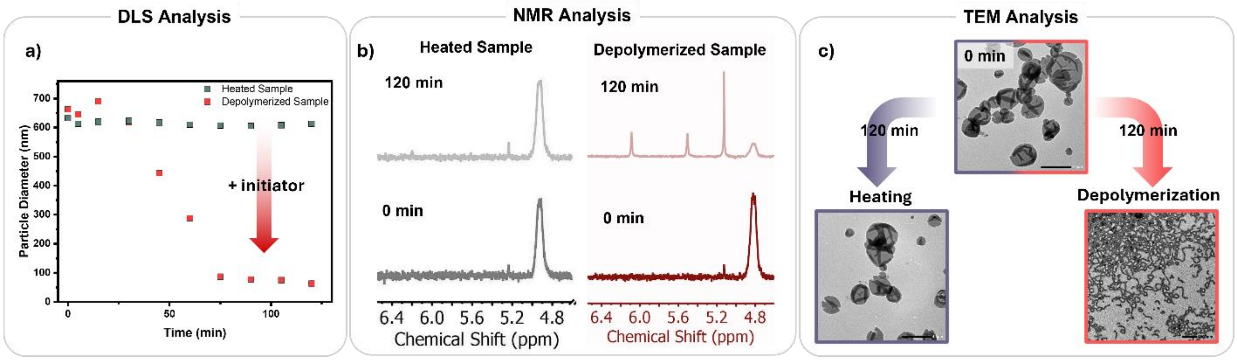

Figure S7: (a) Change in nanoparticle diameter observed upon thermal annealing alone (black data) and depolymerization in the presence of additional initiator (red data). (b) ^{1}H NMR spectra recorded at 0 min and 120 min for the corresponding thermally annealed sample (grey, 0% BzMA regeneration) and the depolymerized sample (33% BzMA regeneration). (c) TEM analysis showing the initial morphology (vesicles, scale bar = 1 µm), the morphology obtained after heating to 100 °C for 120 min (vesicles, scale bar = 1 µm), and the morphology obtained after depolymerization for 120 min at 100 °C (worms, scale bar = 200 nm).

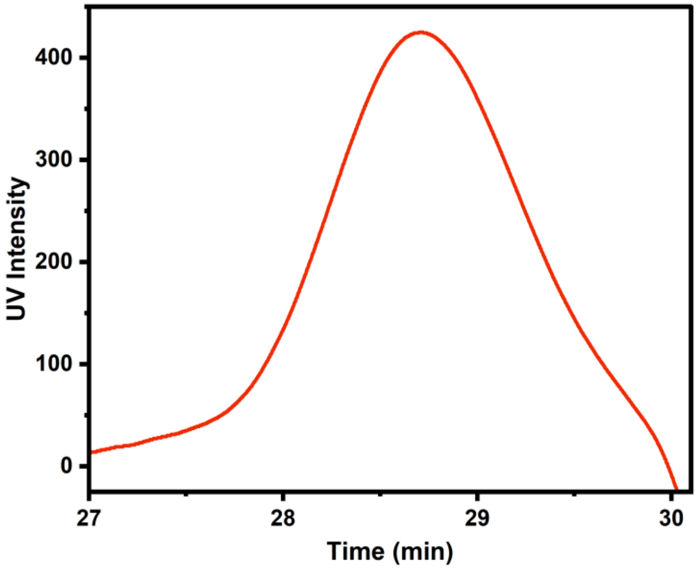

Figure S8: UV SEC trace recorded after 40% BzMA regeneration for $PLMA_{16}$-$PBzMA_{72}$ vesicles heated at 100 °C for 120 min. The strong UV signal observed at 310 nm confirms retention of the RAFT end-group on the copolymer chains at this time point.

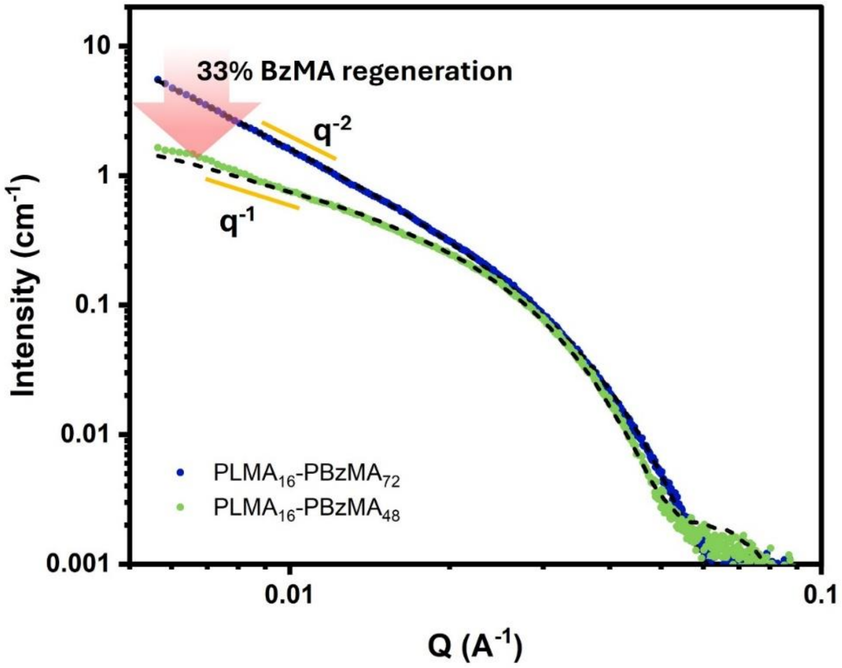

Figure S9: Changes in the SAXS intensity between PLMA$_{16}$-*b*-PBzMA$_{72}$ (starting polymer with vesicular morphology) and PLMA$_{16}$-*b*-PBzMA$_{48}$ (after 33% monomer regeneration with worm morphology).

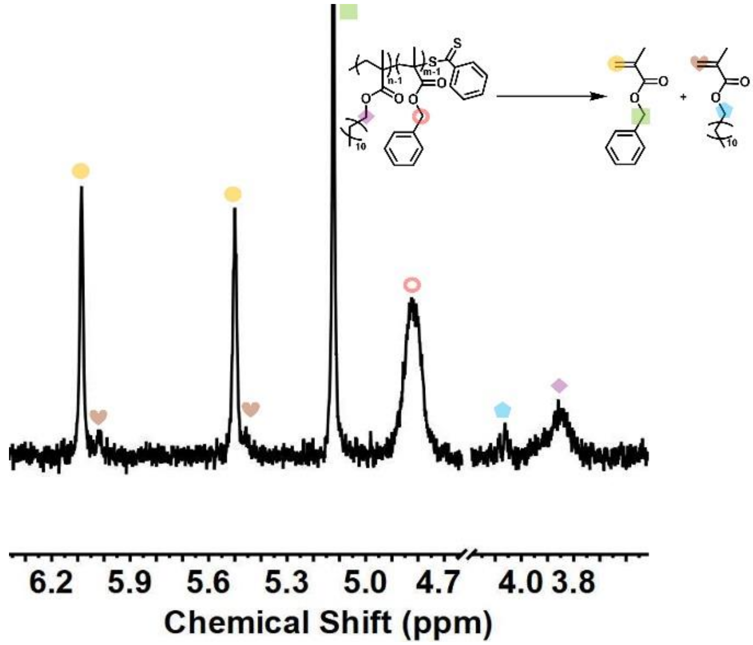

Figure S10: ^{1}H-NMR spectrum at 150 minutes of depolymerization of PLMA$_{16}$-*b*-PBzMA$_{72}$ showing LMA vinyl (≈5.5 and 6.0 ppm) and methylene (≈4.1 ppm) peaks, along with BzMA vinyl (≈5.6 and 6.2 ppm) and benzylic (≈4.8 ppm) peaks.

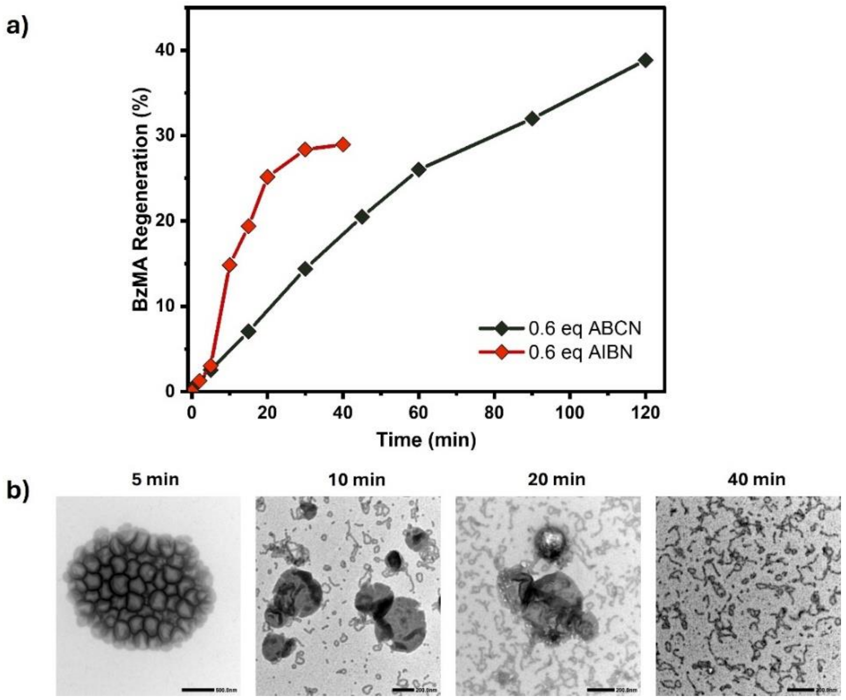

Figure S11: a) Kinetics of formation of BzMA monomer using 0.6 equivalents of ABCN and AIBN, b). Morphological transformation in the presence of 0.6 equivalents of AIBN at 5 min (vesicles, scale bar = 200 nm), 10 min (vesicles+worms, scale bar = 200 nm), 20 min (vesicles+worms, scale bar = 200 nm), 40 min (worms, scale bar = 200 nm).

3.1.2 Vesicles + Worms to Spheres Transformation using $PLMA_{16}$-*b*-$PBzMA_{56}$

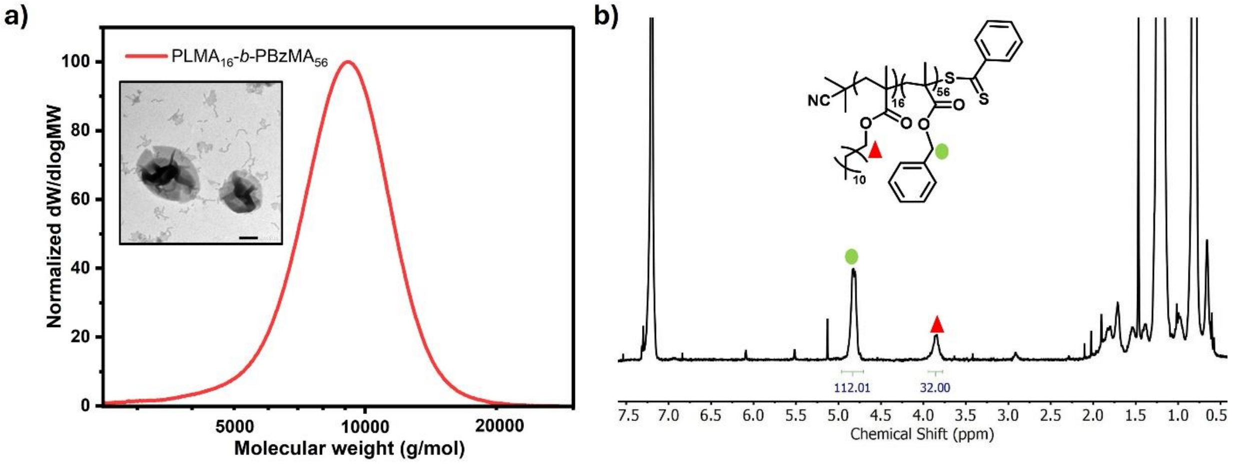

Figure S12: Characterization of $PLMA_{16}$-*b*-$PBzMA_{56}$ polymerization a) SEC trace (M_n = 9600, Đ = 1.1), inset: TEM image of obtained morphology (vesicles + worms), scale bar = 200 nm. b) ^{1}H-NMR spectrum for polymerization of

PLMA$_{16}$-*b*-PBzMA$_{56}$ after 96% conversion of BzMA in 20 wt% *n*-dodecane. The exact degree of polymerization (DP) was confirmed by integrating the methylene protons of PLMA at 3.8 ppm, assigning them to 32 protons (16 repeat units × 2 protons), and then integrating the benzylic protons of PBzMA at 4.9 ppm accordingly (112 protons correspond to 56 units of BzMA, as each repeating unit contains 2 benzylic protons).

Table S5: The morphological transformation obtained through depolymerization of PLMA$_{16}$-*b*-PBzMA$_{56}$

Time (min)	Monomer Regeneration (%) BzMA	LMA	[b]Calculated DP of PBzMA$_x$	[c]Observed Morphologies
0	0	0	56	Vesicles+Worms
5	1	0	55	Vesicles+Worms
15	5	0	53	worms
30	9	0	51	Worms
45	13	0	48	Worms
60	19	0	46	Worms
90	23	0	43	Worms
120	30	0	39	Spheres
150	35	0	36	Spheres
180	38	0	35	Spheres

[a] Calculated according to section 2.5. [b] Calculated according to section 2.6. [c] Morphologies were observed by TEM.

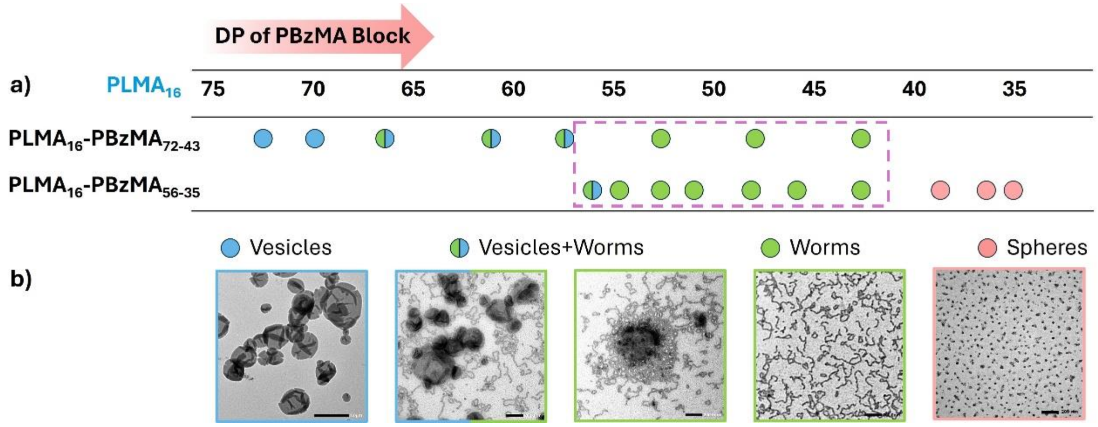

Figure S13: a) Full morphological transformation of PLMA-b-PBzMA polymers with DP 16 stabilizer block. The 1st row displays the morphological transformation starting from PLMA$_{16}$-b-PBzMA$_{72}$. The 2nd row displays the morphological transformation starting from PLMA$_{16}$-b-PBzMA$_{56}$. Purple dotted line indicates overlapping morphologies from both kinetic experiments. b) TEM images of obtained morphologies, scale bars = 1.0 µm (vesicles), 200 nm (vesicles+worms), 200 nm (worms), 200 nm (worms).

3.2 Morphological Transformation of Block Copolymers with $PLMA_{15}$ Stabilizer Block

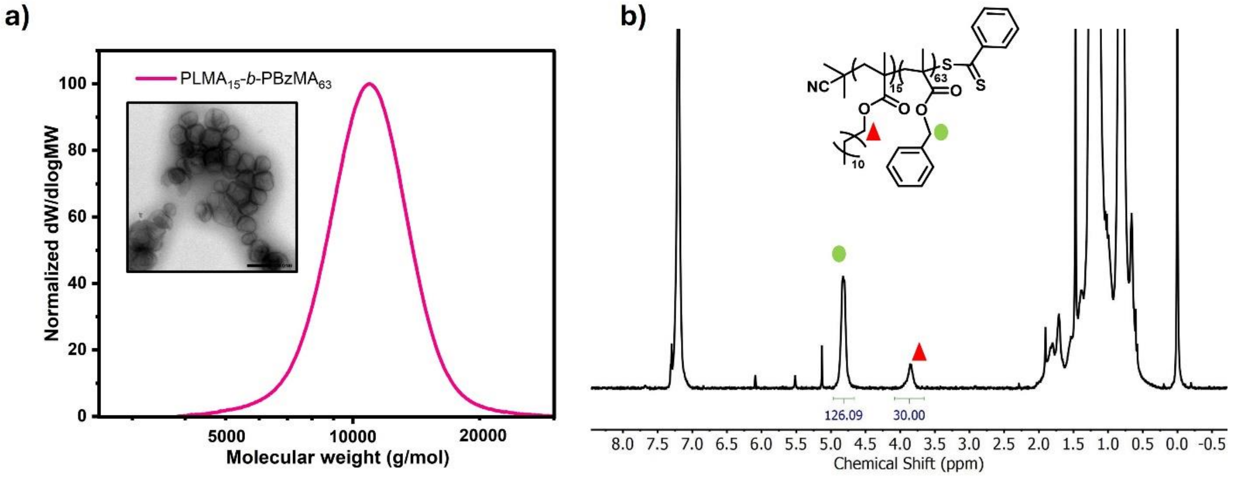

Figure S14: Characterization of $PLMA_{15}$-b-$PBzMA_{63}$ a) SEC trace (M_n=10500, Đ=1.1), inset: TEM image of obtained morphology (vesicles), scale bar = 500 nm. b) ^{1}H-NMR spectrum for $PLMA_{15}$-b-$PBzMA_{63}$ after 95% conversion of BzMA in 20 wt% n-dodecane. The exact degree of polymerization (DP) was confirmed by integrating the methylene protons of PLMA at 3.8 ppm, assigning them to 30 protons (15 repeat units × 2 protons), and then integrating the benzylic protons of PBzMA at 4.9 ppm accordingly (126 protons correspond to 63 units of BzMA, as each repeating unit contains 2 benzylic protons).

Table S6: The morphological transformation obtained through depolymerization of $PLMA_{15}$-*b*-$PBzMA_{63}$.

**Time (min)**	**Monomer Regeneration (%)**		**[b]Calculated DP of $PBzMA_x$**	**[c]Observed Morphologies**
	**BzMA[a]**	**LMA**		
0	0	0	63	Vesicles
5	3	0	61	Vesicles+Worms
15	6	0	59	Vesicles+Worms
30	19	0	51	Worms
45	21	0	49	Worms
60	25	0	47	Worms
75	32	0	43	Worms
90	36	0	39	Spheres
105	41	0	37	Spheres
120	43	0	36	Spheres

[a] Calculated according to section 2.5. [b] Calculated according to section 2.6. [c] Morphologies were observed by TEM.

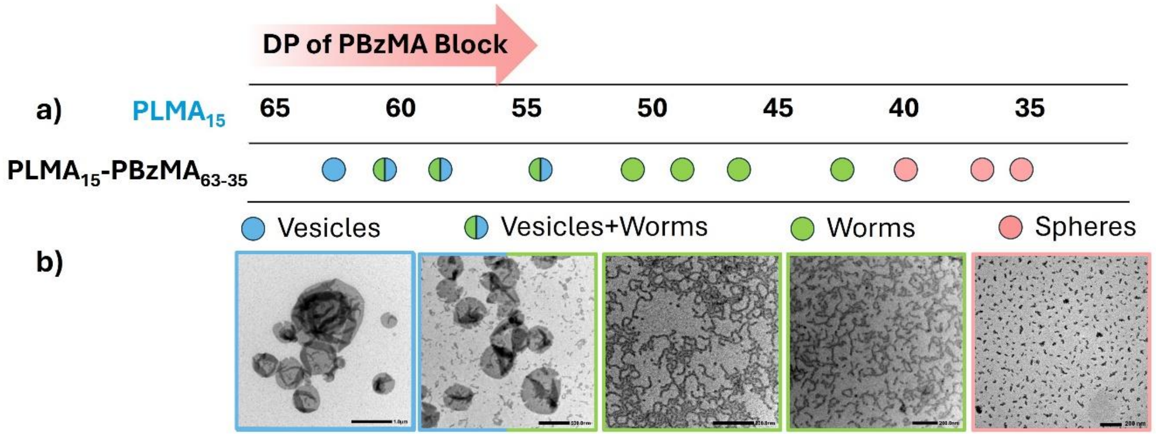

Figure S15: a) Full morphological transformation of PLMA-*b*-PBzMA polymers with DP 15 stabilizer block. b) TEM images of obtained morphologies, scale bars = 1.0 µm (vesicles), 500 nm (vesicles+worms), 200 nm (worms), 200 nm (spheres).

3.3 Morphological Transformation of Block Copolymers with $PLMA_{14}$ Stabilizer Block

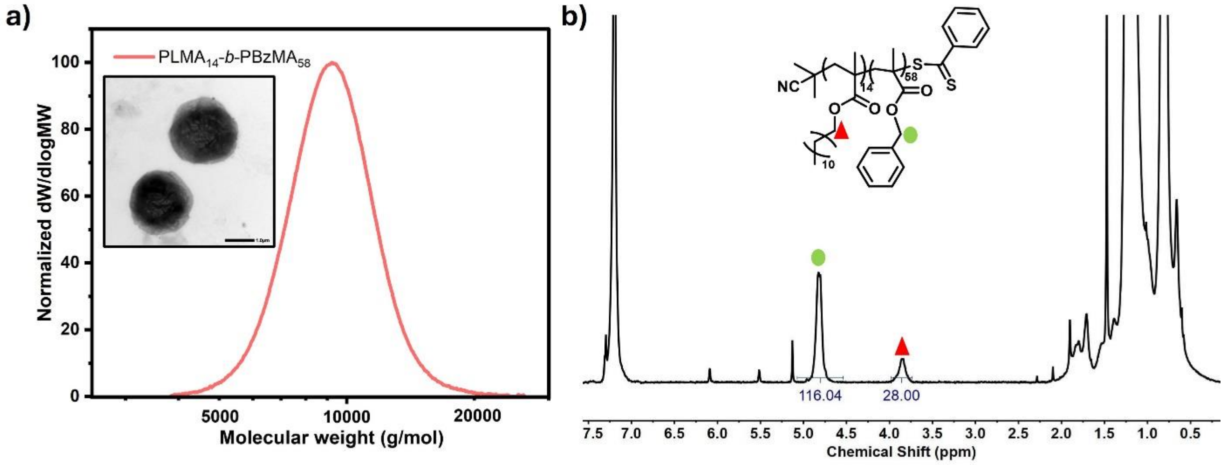

Figure S16: Characterization of $PLMA_{14}$-*b*-$PBzMA_{58}$ a) SEC trace (M_n=8900, Đ=1.1), inset: TEM image of obtained morphology (vesicles), scale bar = 1.0 µm. b) ^{1}H-NMR spectrum for $PLMA_{14}$-*b*-$PBzMA_{58}$ after 95% conversion of BzMA in 20 wt% *n*-dodecane. The exact degree of polymerization (DP) was confirmed by integrating the methylene protons of PLMA at 3.8 ppm, assigning them to 28 protons (14 repeat units × 2 protons), and then integrating the benzylic protons of PBzMA at 4.9 ppm accordingly (116 protons correspond to 58 units of BzMA, as each repeating unit contains 2 benzylic protons).

Table S7: The morphological transformation obtained through depolymerization of $PLMA_{14}$-*b*-$PBzMA_{58}$.

Time (min)	Monomer Regeneration (%) BzMA[a]	LMA	[b]Calculated DP of $PBzMA_x$	[c]Observed Morphologies
0	0	0	58	Vesicles
5	1	0	57	vesicles

15	7	0	54	Vesicles+Worms
30	17	0	48	Worms
45	29	0	41	Worms
60	32	0	38	Spheres
75	44	0	32	Spheres

[a] Calculated according to section 2.5. [b] Calculated according to section 2.6. [c] Morphologies were observed by TEM.

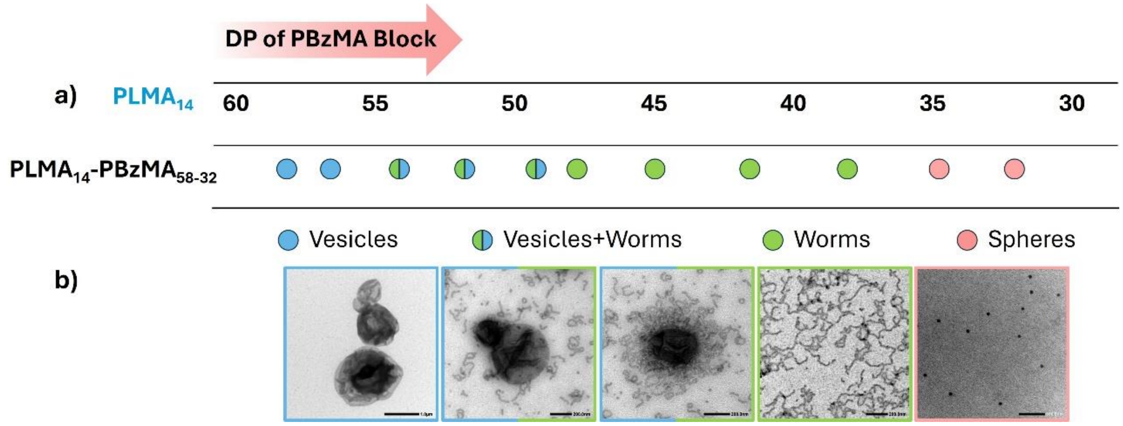

Figure S17: a) Full morphological transformation of PLMA-b-PBzMA polymers with DP 14 stabilizer block. b) TEM images of obtained morphologies, scale bars = 1.0 µm (vesicles), 200 nm (vesicles+worms), 200 nm (worms), 100 nm (spheres).

3.4 Additional Kinetic Experiments to Complete the Phase Diagram

Table S8: The morphological transformation obtained through depolymerization of PLMA$_{16}$-*b*-PBzMA$_{83}$.

Time (min)	Monomer Regeneration (%)		[b]Calculated DP of PBzMA$_x$	[c]Observed Morphologies
	BzMA[a]	LMA		
0	0	0	83	vesicles
5	2	0	80	vesicles
15	13	0	71	vesicles
30	16	0	69	vesicles + worms
45	18	0	67	vesicles +worms
60	19	0	66	vesicles + worms
90	20	0	65	vesicles + worms
120	26	0	60	vesicles + worms
150	34	0	55	vesicles + worms

[a] Calculated according to section 2.5. [b] Calculated according to section 2.6. [c] Morphologies were observed by TEM.

Table S9: The morphological transformation obtained through depolymerization of $PLMA_{15}$-*b*-$PBzMA_{76}$.

Time (min)	Monomer Regeneration (%) BzMA	LMA	[b]Calculated DP of $PBzMA_x$	[c]Observed Morphologies
0	0	0	76	vesicles
5	3	0	74	vesicles
15	9	0	69	vesicles
30	15	0	65	vesicles
45	20	0	61	vesicles +worms
60	24	0	58	vesicles + worms
90	30	0	53	vesicles + worms
120	40	0	46	worms

[a] Calculated according to section 2.5. [b] Calculated according to section 2.6. [c] Morphologies were observed by TEM.

Table S 10: The morphological transformation obtained through depolymerization of $PLMA_{14}$-b-$PBzMA_{76}$.

Time (min)	Monomer Regeneration (%) BzMA	LMA	[b]Calculated DP of $PBzMA_x$	[c]Observed Morphologies
0	0	0	76	vesicles
5	1	0	75	vesicles
15	8	0	70	vesicles
30	18	0	62	vesicles
45	24	0	58	vesicles
60	27	0	56	vesicles
90	33	0	51	vesicles+worms
120	35	0	49	vesicles+worms

[a] Calculated according to section 2.5. [b] Calculated according to section 2.6. [c] Morphologies were observed by TEM.

3.5 Depolymerization of PLMA-b-PBzMA System with Longer Stabilizer Block ($PLMA_{22}$)

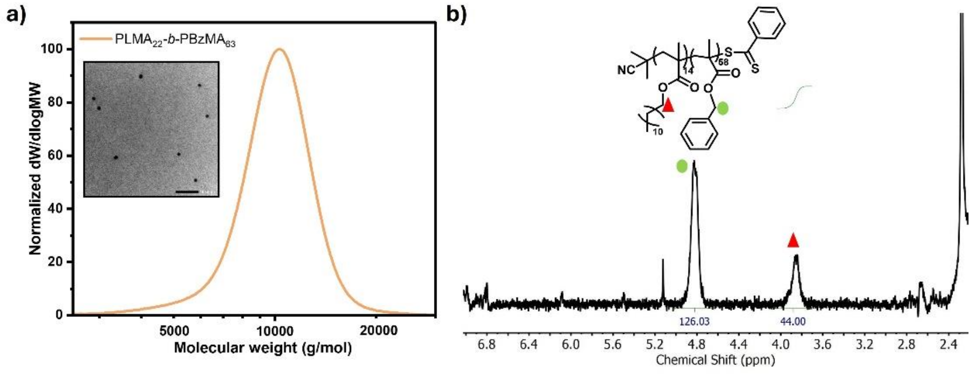

Figure S18: Characterization of $PLMA_{22}$-b-$PBzMA_{63}$ a) SEC trace (M_n=9500, Đ=1.1), inset: TEM image of obtained morphology (vesicles), scale bar = 100 nm. b) ^{1}H-NMR spectrum for $PLMA_{14}$-b-$PBzMA_{58}$ after 95% conversion of

BzMA in 20 wt% n-dodecane. The exact degree of polymerization (DP) was confirmed by integrating the methylene protons of PLMA at 3.8 ppm, assigning them to 44 protons (14 repeat units × 2 protons), and then integrating the benzylic protons of PBzMA at 4.9 ppm accordingly (126 protons correspond to 63 units of BzMA, as each repeating unit contains 2 benzylic protons).

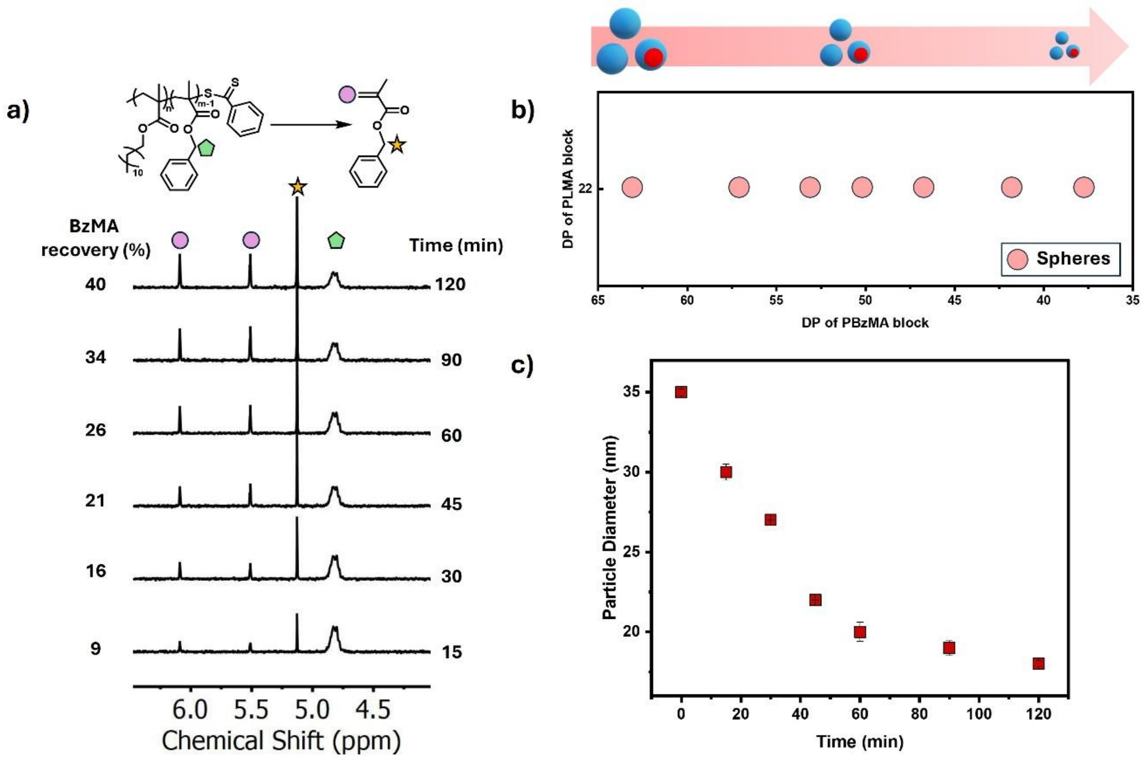

Figure S19: Depolymerization of the core-forming block for PLMA22-b-PBzMA63 spherical nanoparticles monitored by: (a) ^{1}H NMR spectroscopy; (b) morphological transformation observed through TEM analysis; and (c) particle size evolution observed by DLS analysis.

Table S11*:* The morphological transformation obtained through depolymerization of $PLMA_{22}$-*b*-$PBzMA_{63}$.

Time (min)	Monomer Regeneration (%)		[b]Calculated DP of $PBzMA_x$	[c]Observed Morphologies
	BzMA	LMA		
0	0	0	63	Spheres
15	9	0	57	Spheres
30	16	0	53	Spheres
45	21	0	50	Spheres
60	26	0	47	Spheres
90	34	0	42	Spheres
120	40	0	38	Spheres

[a] Calculated according to section 2.5. [b] Calculated according to section 2.6. [c] Morphologies were observed by TEM.

3.6 Polymerization vs Depolymerization

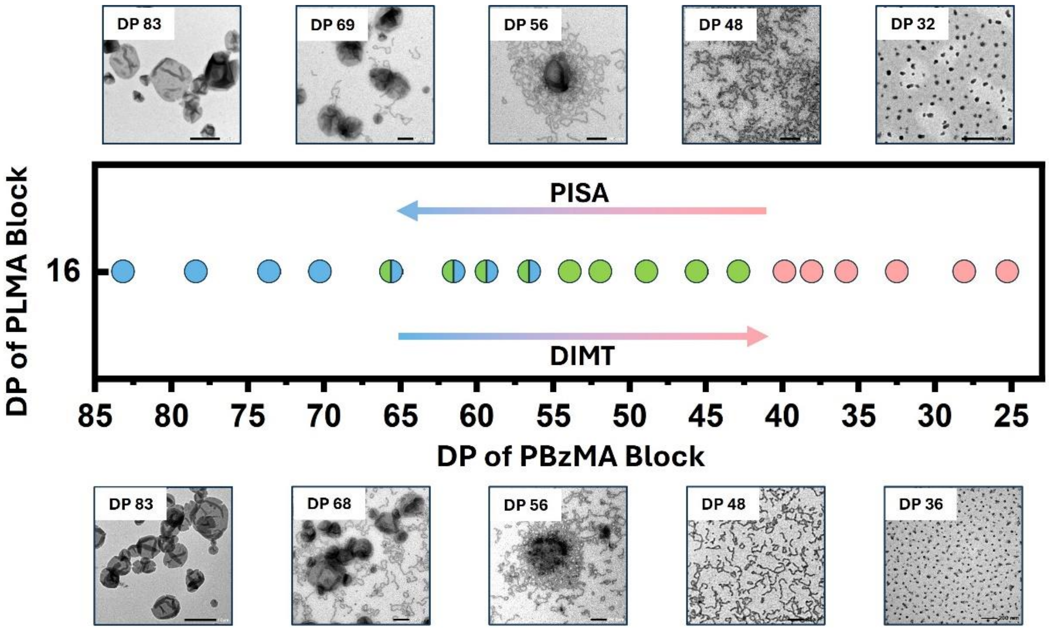

Figure S20: Comparison of phase diagrams of the PISA (top) and DIMT (bottom) processes for PLMA-*b*-PBzMA block polymers containing 16 LMA units. PISA was performed at 20 wt% solids in *n*-dodecane at 80 °C, while depolymerization was carried out at 0.7 wt% solids (25 mM RUC) in *n*-dodecane at 100 °C with 0.6 equivalents of ABCN. TEM images have scale bars of 500 nm (vesicles, PISA), 200 nm (vesicles + worms, PISA), 200 nm (worms, PISA), 200 nm (spheres, PISA), 1.0 µm (vesicles, DIMT), 200 nm (vesicles + worms, DIMT), 200 nm (worms,DIMT), 200 nm (spheres, DIMT).

3.7 Demonstration of Reversibility via an In Situ Depolymerization–Repolymerization Cycle

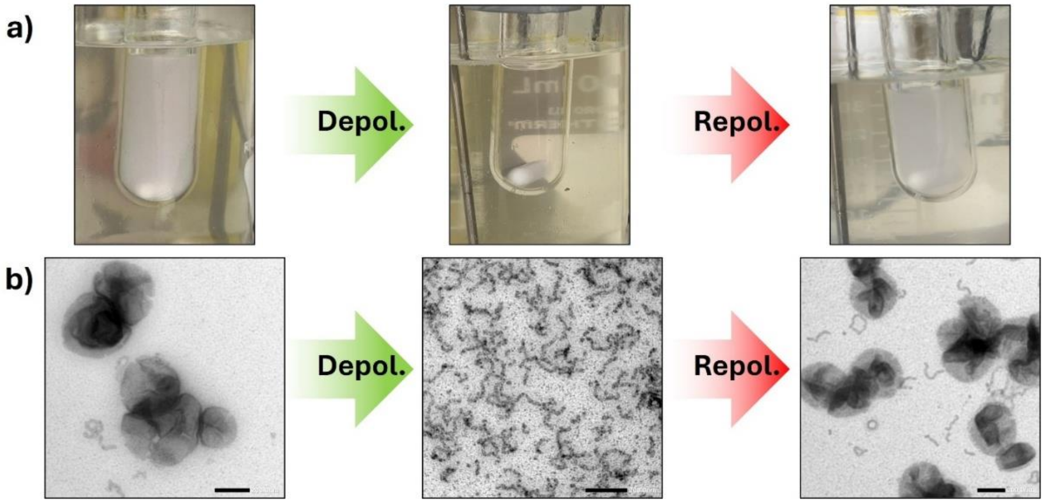

Figure S21: (a) Digital photographs recording the visual appearance of the reaction mixture during depolymerization at 100 °C (using 0.4 equivalents ABCN, 25 mM RUC, 90 min, 25% BzMA release) and repolymerization (using 0.2

equivalents AIBN, 25mM RUC, 80°C, 25 µL BzMA, 240 min, 17% BzMA incorporation). (b) Corresponding morphological evolution monitored by TEM analysis during both processes.

3.8 Isolation of Nanoparticles

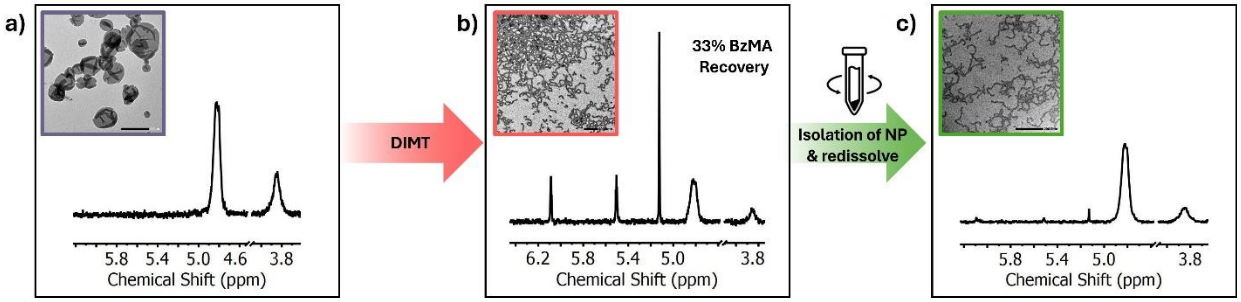

Figure S22: TEM images and ^{1}H-NMR spectra recorded for (a) initial PLMA16-*b*-PBzMA72 vesicles, TEM image scale bar = 1.0 µm, (b) depolymerized PLMA16-*b*-PBzMA48 worms, TEM image scale bar = 200 nm, and (c) PLMA16-*b*-PBzMA48 worms isolated via centrifugation to remove the regenerated BzMA monomer, TEM image scale bar = 200 nm.

3.9 Tube Inversion Test

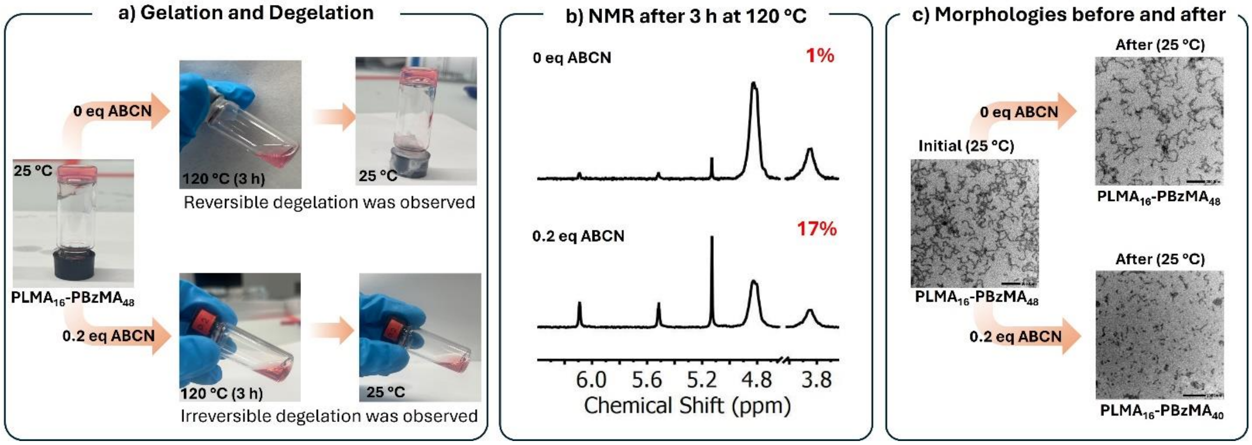

Figure S23: DIMT as a tool for controlling the degelation of PLMA-*b*-PBzMA polymers with 12 wt% of PLMA16-*b*-PBzMA48 polymers. a) Tube inversion test of PLMA16-*b*-PBzMA48 with 0 equivalents of ABCN (top), and 0.2 equivalents of ABCN (bottom), b) ^{1}H-NMR spectra and monomer conversions after heating at 120 °C for 3 h with 0 equivalents of ABCN (top), and 0.2 equivalents of ABCN (bottom), c) TEM images of the morphologies at 25 °C before and after cooling down, followed by heating at 120 °C (cooled down to 25 °C) for 3 h with 0 equiv. of ABCN (top), and 0.2 equiv. of ABCN (bottom), scale bar = 200 nm.

4 References

(1) Fielding, L. A.; Lane, J. A.; Derry, M. J.; Mykhaylyk, O. O.; Armes, S. P. Thermo-responsive diblock copolymer worm gels in non-polar solvents. *J. Am. Chem. Soc.* **2014**, *136* (15), 5790-5798.

(2) Perrier, S. 50th Anniversary Perspective: RAFT Polymerization A User Guide. *Macromolecules* **2017**, *50* (19), 7433-7447.
(3) Sigma-Aldrich. *Thermal Initiators: Decomposition Rate and Half-Life*. Sigma-Aldrich, https://www.sigmaaldrich.com/deepweb/assets/sigmaaldrich/marketing/global/documents/411/888/thermal_initiators.pdf?msockid=2f1f9f5bf9a963e50d8d8968f813620e (accessed 2025 August 05).
